# The Genus *Plagiothecium* Schimp. (Plagiotheciaceae, Bryophyta) in Eurasia: An Annotated Checklist with Distribution and Ecological Data

**DOI:** 10.3390/plants10050868

**Published:** 2021-04-26

**Authors:** Grzegorz J. Wolski, Samar Nour-El-Deen, Alicja Cienkowska, Daniel Bożyk, Wagieh El-Saadawi

**Affiliations:** 1 Department of Geobotany and Plant Ecology, Faculty of Biology and Environmental Protection, University of Lodz, Banacha 12/16, 90-237 Łódź, Poland; grzegorz.wolski@biol.uni.lodz.pl (G.J.W.); alicja.cienkowska@unilodz.eu (A.C.); daniel.bozyk@unilodz.eu (D.B.); 2 El-Saadawi Lab, Department of Botany, Faculty of Science, Ain Shams University, Cairo 11566, Egypt; wagelsaadawi@yahoo.com

**Keywords:** checklist, ecological preferences, Eurasia, geographic distribution, Musci, *Plagiothecium*

## Abstract

An annotated checklist of the pleurocarpous moss genus *Plagiothecium* in Eurasia is presented for the first time based on a thorough review of the literature. Data have been compiled from previous relevant works conducted on the genus over more than 70 years and published up to the end of June 2020 for 107 Eurasian countries (and islands). Sectional classification is based on molecular phylogeny of the genus published recently. A total of 41 taxa are reported, including 29 species and 12 infraspecific taxa (nine varieties and three forms) belonging to eight sections. The highest numbers of taxa were found in China (20 taxa), the Russian Federation (20 taxa) and Japan (18 taxa), while the smallest numbers of taxa were recorded in the Middle East, Central Asia and the islands area. Not a single species of *Plagiothecium* was recorded in 26 regions, whereas *P. denticulatum*, *P. nemorale* and *P. cavifolium* turned out to be the most widespread species in the entire study area. They were recorded in most of the surveyed countries and islands. For each accepted taxon, information on relevant literature, synonyms, distribution within Eurasia and globally are provided. Comments on each taxon, ecological preferences, and notes on doubtful records are also included. Additionally, distribution maps for each recognised taxon are supplied. This checklist can enlighten and foster a better understanding of the distribution, diversity, and ecology of *Plagiothecium* in Eurasia and provides an incentive for future research on the genus.

## 1. Introduction

The pleurocarpous moss genus *Plagiothecium* Schimp. (Plagiotheciaceae M. Fleisch.) was erected by Schimper and initially described in “*Bryologia Europaea*” [1] to include plants with more or less flattened, asymmetrical leaves, well-developed decurrent alar cells, and double costae. Over time, the understanding of this genus changed, and many species were excluded from it to create new genera, viz. *Herzogiella* Broth., *Isopterygiella* Ignatov & Ignatova, *Isopterygiopsis* Z. Iwats., *Isopterygium* Mitt., *Pseudotaxiphyllum* Z. Iwats. and *Taxiphyllum* M. Fleisch. Our understanding of the family Plagiotheciaceae has also changed. It has been treated for a long time as monogeneric, with the single genus *Plagiothecium* [2,3,4], but further research using molecular methods [5,6,7] changed the view of the described genus and the whole family.

Biogeographically, *Plagiothecium* is a cosmopolitan genus represented in all continents, though varies in diversity and abundance, with largest concentrations in temperate, upland habitats of the Northern Hemisphere. Apart from Asia and Europe [8,9] (which will be discussed in detail later in this paper), the genus has been reported from, for example, **Africa**: Sub-Saharan Africa [10], tropical Africa [11], Northern Africa [12] and Western Africa (Equatorial Guinea) [13]; **Antarctica**: [14]; **Subantarctica**: [15]; **Australasia**: [11,16]; **Australia**: a single record according to Klazenga [17]; **North America**: [18,19]; **North America North of Mexico**: [20,21]; **South America**: Tropical Andean countries [22]; and **Latin America** [3].

**Figure 1:**
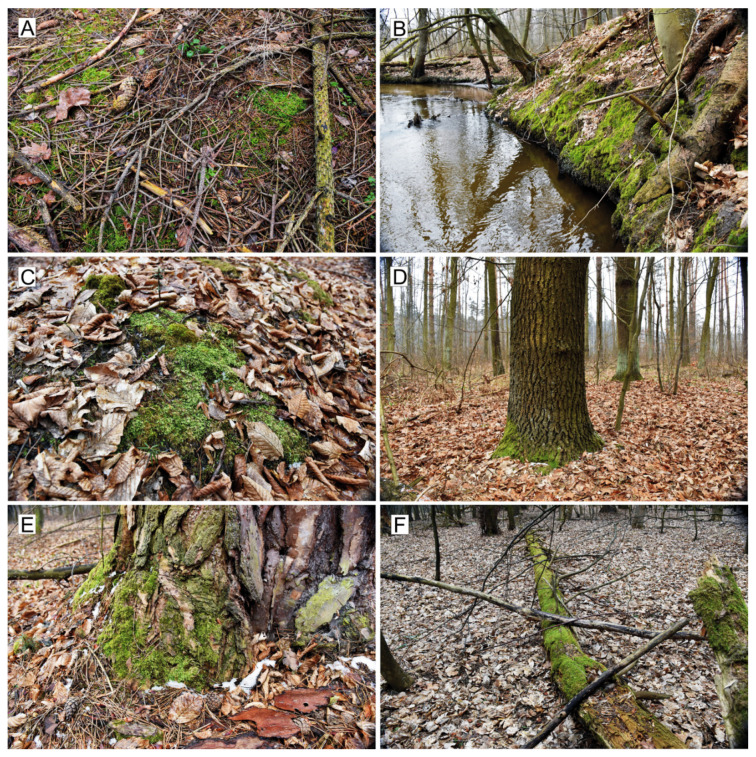
Examples of habitats and substrates covered in central Europe by *Plagiothecium* species. (**A**–**C**). Epigeic habitat: (**A**). *P*. *curvifolium* in the coniferous forest with *Picea abies*, (**B**). *P*. *angusticellum* and *P*. *longisetum* on the river side in the *Fraxino-Alnetum* phytocoenosis; (**C**). *P*. *denticulatum* in the deciduous forest with *Fagus sylvatica*; (**D**,**E**). Epiphytic habitats: (**D**). *P*. *denticulatum* and *P*. *curvifolium* on the lowest part of the trunk of *Quercus robur;* (**E**). *P*. *curvifolium* on the trunk of *Pinus sylvestris*); (**F**). Epixylic habitats: *P*. *laetum* on a log in a deciduous forest (photos by G.J. Wolski, 14 March 2021, Central Poland).

Ecologically, *Plagiothecium* is mainly terrestrial, subaquatic, or aquatic; inhabits a variety of substrata, i.e., epilithic (on stones and rocks usually covered with a layer of humus), epixylic (on wood), epigeic (on soil) or epiphytic (usually on the lowest part of tree trunk), see, Figure 1. Species of the genus grow in shaded, moist places in forests, and also found in montane and exposed habitats. They occur mostly in temperate and boreal zones and in tropical uplands; occasionally found in dry and lowland tropics [3,23].

So far many new taxa have been globally described within the genus; however, the total number of accepted species is still uncertain [24]. Estimates of the number of species have changed over time—from 80–90 species [3] up to 110 species [25]. The latest taxonomic research based on molecular analyses confirmed 70 taxa worldwide [7,26,27], with additional 46 taxa awaiting detailed research to determine their taxonomic status [7].

Since the mid-twentieth century, the number of species of the genus *Plagiothecium* given for Asia and Europe remained unchanged. For each of these continents, it remained at a similar, fairly low level, containing from a few to over a dozen species [28,29,30,31,32,33]. This fact was the result of little scientific interest in the described genus which for the past 50 years was rarely the subject of any specified studies, which were usually carried out on national or local level [18,19,29,30]. This situation has started to change over the past few years [7,23,26,27].

The genus *Plagiothecium* is usually described as very variable; however, the reasons for this variability were not analysed [25,28,29,30,34]. Taxonomic studies combined with molecular analyses allowed us not only to describe a number of new taxa but also provided a new perspective on interspecies relationships within the genus. Thanks to this type of research, it has been pointed out that many species are complexes, and the high variability of individual species has been explained [7,23,26,27,35,36,37].

Currently, research on the genus *Plagiothecium* focused mainly on the taxonomy of individual taxa or of the whole genus [7,23,26,27]. Other aspects as physiology, ecology or chorology were examined in the previous century [38,39,40,41,42] and were usually included as appendices to taxonomic research, i.e., not the main subject of researchers’ interest.

The present study was carried out due to the fact that the distribution of *Plagiothecium* taxa has never been the subject of specific studies and the fact that in recent years the taxonomic status of many species has changed. The purpose of this article is to create a checklist for *Plagiothecium* in Eurasia and to describe the distribution of the Eurasian taxa of this genus. To attain this goal, it was decided to amass and systematise the available knowledge in relevant literature in order to (1) identify the names of all taxa known to occur in each country (island), (2) clarify the status of names and taxa which are accepted, (3) analyse the geographic distribution, and (4) estimate species richness of *Plagiothecium* in Eurasia. This study is not intended to be a taxonomic revision of *Plagiothecium*, however it provides the latest status for all recognised taxa with their geographical distribution.

We tried our utmost to compile all of the relevant information to approach the purpose for this study. However, the current knowledge of *Plagiothecium* distribution and species diversity in Eurasia (and other continents as well) is still somewhat biased toward some countries while contains gaps in others due to complete lack of data or of reliable data or insufficient sampling. Not to mention the currently accelerating rate of changes in taxonomic status of many taxa as a consequence of molecular studies. The present checklist can serve as a new starting point for further research on the genus in these countries, which will likely result in addition of new records or perhaps new taxa.

The authors inviting comments and would appreciate notifications of additions and corrections to update the present checklist. Please address relevant information to the corresponding author.

## 2. Results and Discussion

### 2.1. General Results

The present checklist includes 41 taxa, comprising 29 species, nine varieties and three forms belonging to eight sections. For two countries (Palestine and Timor-Leste), no study confirming or excluding any species from the genus described could be found. The rest of Eurasia is quite well explored and widely described in terms of mosses (Table 1).

All across Eurasia, the genus *Plagiothecium* is quite widely, but unevenly distributed (Figures S1–S4). An analysis of the species richness for individual countries and islands has shown that in the area of the Middle East (e.g., Iraq, Israel, Jordan, Kuwait, Lebanon and Qatar), Central Asia (Kazakhstan, Kyrgyzstan, Tajikistan, Turkmenistan and Uzbekistan), and small islands (e.g., the Azores, Balearic Islands, Canary Islands, as well as Crete, Malta and Sardinia) taxa from the genus *Plagiothecium* were not recorded at all or are sporadically recorded (Figures S2 and S3). Further analysis also showed that in 26 countries (25% of all analysed countries) not a single taxon of the concerned genus was recorded. Only one species was recorded in 11 countries (8%), two and three taxa were recorded in six regions (6% among all analysed countries and islands) (Table 2).

The highest number of taxa was recorded in eight quite large countries (8% among all analysed): in China (20 taxa), the Russian Federation (20), Japan (18); Austria, Germany, Sweden (16), Czech Republic and Switzerland (15 taxa each) (Table 2, Figures S1–S4).

The conducted analysis of the frequency of occurrence of individual species showed that species with a limited range are a majority in the studied genus. Twenty-six taxa (60% of all) were reported only from one to five countries, and in the Eurasia they were recognised to be very rare. Wherein, 12 of them (e.g., *P*. *argentatum*, *P*. *cochleatum*, *P*. *conostegium*, *P*. *decoratum*, *P*. *subglaucum*) (Figure S1) were listed only in Asia. The smallest number of taxa in this group (*P*. *berggrenianum*, *P*. *enerve*, *P*. *fallax*, *P*. *obtusissimum* and *P*. *rossicum*) was reported both in Asia and Europe (Table 2). The frequency analysis also indicated that common species are the least numerous group. The species with the widest range were: *P*. *denticulatum*, listed in 65 countries and islands (65% of all analysed), *P*. *nemorale* (64, 62%), and *P*. *cavifolium* (59, 60%), they were considered as common in the study area (Table 2, Figures S2 and S4).

### 2.2. Annotations by Taxon

**Figure 2:**
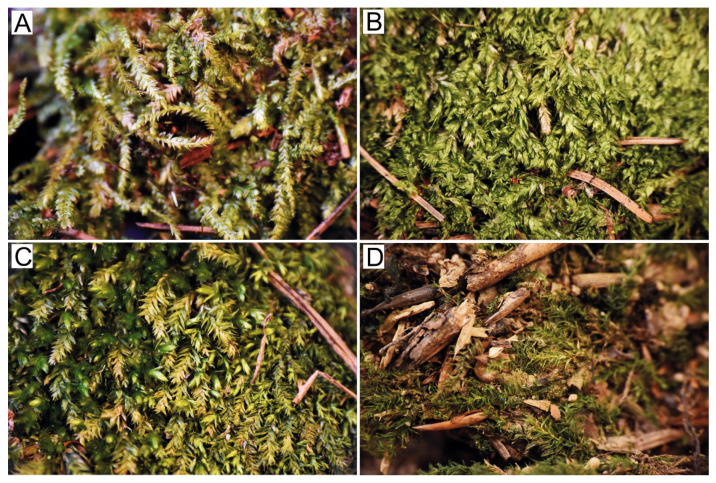
Arrangement of the leaves on the stem. (**A**). Flattened and glossy leaves of *P. curvifolium*; (**B**). Imbricate leaves of *P. denticulatum*; (**C**). Julaceus leaves of *P. cavifolium* (dark green) and flattened, imbricate leaves of *P. longisetum* (yellowish); (**D**). Shrunken, dark green leaves of *P. nemorale* (photos by G.J. Wolski, 15 March 2021).

**Figure 3:**
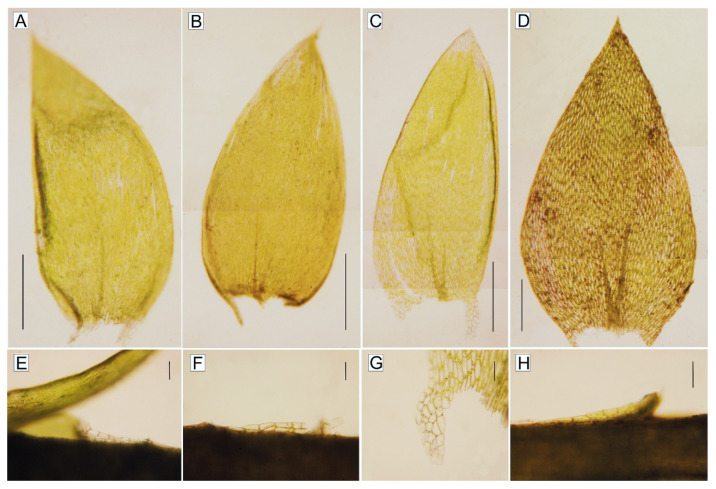
Leaves and decurrent angular cells of selected *Plagiothecium* species. (**A**–**D**). Shape and leaf symmetry: (**A**). *P*. *curvifolium*; (**B**). *P*. *cavifolium*; (**C**). *P*. *denticulatum*; (**D**). *P*. *nemorale* (scale 500 µm); (**E**–**H**). Shape and dimensions of the decurrent cells: (**E**). *P*. *curvifolium*; (**F**). *P*. *cavifolium*; (**G**). *P*. *denticulatum*; (**H**). *P*. *nemorale* (scale 100 µm) (photos by G.J. Wolski, 15 March 2021).

**Figure 4:**
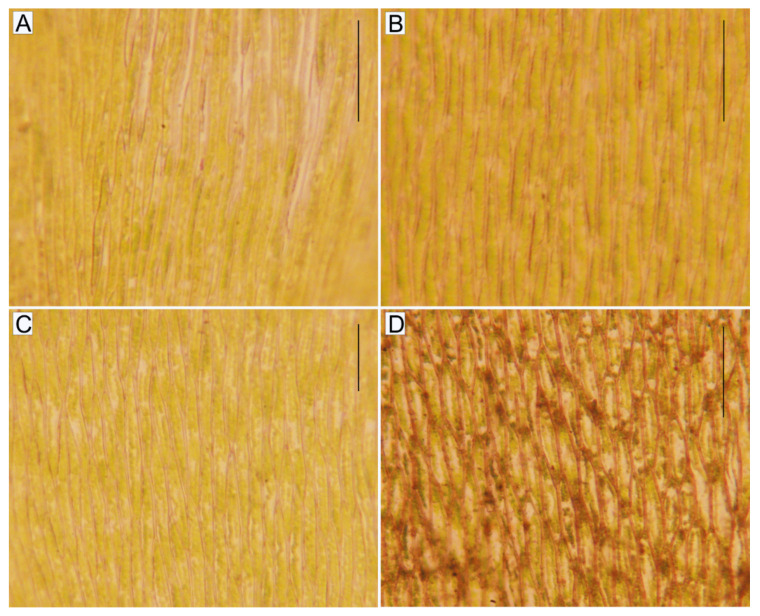
The shape and dimensions of the central part of the leaf cells. (**A**). *P*. *curvifolium*; (**B**). *P*. *cavifolium*; (**C**). *P*. *denticulatum*; (**D**). *P*. *nemorale* (scale 100 µm) (photos by G.J. Wolski, 15 March 2021).

- Annotations to the following list of *Plagiothecium* taxa include brief description for each taxon, with comments on nomenclature and taxonomic status (when necessary), ecological preferences, and geographic distribution inside and outside Eurasia (if available).
- Taxon distribution per country in Eurasia is given in detail in Appendix B, with all available literature sources.
- We did not make any broad attempt to provide an exhaustive list of records for all countries in each continent (outside Eurasia), but we gave a general overview of the geographic distribution outside the study area based on the selected references. As far as we know, none of the 41 taxa listed here have been reported from Antarctica [14] or Australia [17].

1. *Plagiothecium angusticellum* G.J. Wolski & P. Nowicka-Krawczyk 2020—a medium-sized plant, with asymmetrical leaves, not shrunken when dry; an acuminate, gently curved, not serrated apex; tight areolation, created with long and narrow leaf cells which do not form regular rows. This combination of features made it possible to distinguish *P*. *angusticellum* from similar and closely related species such as *P*. *cavifolium*, *P*. *nemorale*, and *P*. *longisetum* [27]. **Ecology**. From Poland (from where it was described), this species was recorded mainly in deciduous forests (e.g., eutrophic swamp forests *Ribeso nigri*-*Alnetum* Sol.-Górn. (1975) 1987, wet alder riparian forests *Fraxino*-*Alnetum* W. Mat. 1952, beech forests *Luzulo pilosae*-*Fagetum* W. Mat. et A. Mat. 1973, oaklinden-hornbeam forests *Tilio*-*Carpinetum* Tracz. 1962) in epigeic, epiphytic and epilithic habitats. **Distribution**. Apart from Central Europe, this species is also reported from North America (USA) [191].
2. *Plagiothecium argentatum* (Mitt.) Q. Zuo 2011—described as *Hypnum argentatum* by Mitten [192], transferred to the genus *Struckia* by Müller [193], and finally (based on DNA analysis) incorporated into the genus *Plagiothecium* [36], its integration in this genus was confirmed by other researchers (e.g., [7,23]). A pale green, julaceous plant, with symmetric, concave, plicate, long, non-decurrent leaves, with a serrulate or entire margin, and quadrate alars; it is characterised by quite an unusual (for a member of the genus *Plagiothecium*) set of features. Most of these features place *P*. *argentatum* well in the described genus, but serrulate margins, absence of decurrency and quadrate alars seem to exclude this species from the genus *Plagiothecium*. This large morphological separateness resulted in the placement of this species (as *P*. *enerve*) in a separate section of *Struckia* [23]. **Ecology**. The species was recorded in epiphytic and epixylic habitats [7]. **Distribution**. So far, recorded from East and South Asia.
3. *Plagiothecium berggrenianum* Frisvoll 1981—a medium-sized to large, julaceous, crowded plant; leaves symmetrical, very concave, ovate, long decurrent, and an abruptly narrowed to hooked apex; leaf cells long and narrow, thick-walled; capsules straight and erect. This circumpolar species was described by Frisvoll [171], and is easily distinguishable from other species by leaves with recurved margins and by the shape of its apex, as well as well-developed alar regions. *Plagiothecium berggrenianum* is similar to *P*. *svalbardense*, but the former is longer, with elliptical, plicate leaves, and broadly recurved margins. Wynns [7] states that *P*. *berggrenianum* is a possible hybrid. **Ecology**. This species is recorded in epigeic, epilithic and epixylic habitats [26,171,172]. It is found in swales, tundra, and cliffs; low to moderate elevations [21]. **Distribution**. Apart from Eurasia, it is also reported from North America (Canada, Greenland, USA) [7,26,172].
4. *Plagiothecium cavifolium* (Brid.) Z. Iwats. 1970—Iwatsuki [29] selected this name for the species previously known as *P*. *roeseanum* [1]. A small to medium-sized, pale green to yellowish green, glossy plant in dense mats (Figure 2); ascending to erect, stems julaceous more or less; imbricate, ovate to elliptical, symmetric, concave leaves (Figure 3), often with a curved apex; cells linear-rhomboidal, long and narrow (Figure 4). **Ecology**. This species is found in shaded locations in low to high elevations [21] and recorded in epigeic, epilithic, epixylic and epiphytic habitats (e.g., [25,28,29,30,34,62,79,194,195,196,197,198,199]). **Distribution**. Apart from Eurasia, this species is also reported from Africa (Tunisia) [47]; North America (Canada, Falkland Islands, Greenland, USA) [7,21,23,25,79].
5. *Plagiothecium cavifolium* var. *orthocladium* (Schimp.) Z. Iwats. 1970—Iwatsuki [29] selected this name for the variety previously known as *P*. *orthocladium* [1], and also reported on a relationship between this taxon and *P*. *nemorale* fo. *japonicum* (currently *P*. *japonicum*) as well as *P*. *succulentum*. Wynns [7] mentioned difficulty in distinguishing this taxon, adding that he used this name for olivaceous, boreal specimens, with crispate and spreading leaves. **Ecology**. This taxon is recorded in epilithic habitat. **Distribution**. Apart from Europe (Nordic countries), this taxon is also reported from North America (Canada, Greenland) [7].
6. *Plagiothecium cochleatum* Dixon 1938—a dark green plant; leaves loosely imbricated, concave, plicate, with rigid areolation, and quite well-developed alar decurrencies. Wynns [7] indicates that *P*. *cochleatum* is similar to *P*. *cavifolium* and may be confused with this species. **Ecology**. It is a rare species, present in disjunct Alpine and Himalayan habitats [23]. **Distribution**. So far, it is reported from Asia (India).
7. *Plagiothecium conostegium* Herzog 1916—Suzuki [119] recorded this taxon from Japan. Considering the features and pictures published by Suzuki [119] in particular: Asymmetric, in dry condition shrunken leaves, long-hexagonal cells, we believe that the taxon described by Suzuki [119] looks more like *P*. *longisetum*. This requires checking herbarium materials, but at this stage, we consider the presence of *P*. *conostegium* doubtful in Eurasia. **Ecology**. In Central and South America, it is recorded as a forest species mostly growing on epigeic, epilithic, epiphytic and epixylic habitats [3,7]. **Distribution**. Apart from Asia (Japan) [119], the species is reported by Wynns [7] as a mountain species from Central America and Northern South America (Bolivia, Ecuador, Guatelama, Mexico, Peru) and also present at high elevations in North America, the Dominican Republic, Northern Andes and Tierra del Fuego [3].
8. *Plagiothecium curvifolium* Schlieph. ex Limpr. 1897—a small to medium-sized plant, green to yellowish green, glossy (Figure 2); leaves broadly lanceolate to lanceolate, not concave, asymmetric, sometimes downward curving; alar decurrencies wide, hyaline (Figure 3), sometimes even inflated; capsules curved and inclined to horizontal. These features distinguish this species from other closely related species (e.g., *P*. *laetum*). Ireland [18] and Iwatsuki [29] (and many after them) did not recognise *P*. *curvifolium* and *P*. *laetum* as separate species, but DNA analysis clearly proves this [7,23]. **Ecology**. It is recorded in epigeic, epilithic, epixylic and epiphytic habitats (e.g., [25,28,29,30,34,79,194,200]). **Distribution**. The species is common in lowland areas [25]. Outside Eurasia, it is reported from Africa [29] but its presence in North Africa considered doubtful by Ros et al. [12]. It is also reported from North America (Canada, USA) [7,25,79].
9. *Plagiothecium curvifolium* fo. *julaceum* Culm. & E. Bauer 1915—it is a forgotten taxon, which after being described in 1915 it was not later mentioned as a separate or at least as a synonym in any of the major bryological studies [28,30,32,201,202,203,204,205]. Based on molecular analyses, Wynns [7] recognises it as separate, although the description of the gametophyte characteristics (*ramis subjulaceis, foliis imbricatis saepe subhomomallis*) indicates features similar to *P*. *cavifolium*. At this stage, this taxon definitely requires further in-depth research and detailed analysis. **Ecology and Distribution**. Detailed ecological data and distribution for this taxon are not known exactly and require specific investigation. However, according to Wynns [7], the isotype is found on fir roots near the upper tree line (epiphytic); from Switzerland (Burgfeld in Beatenberg, Canton of Bern).
10. *Plagiothecium decoratum* J.T. Wynns 2015—the species established by Wynns [23] and described as a julaceous to subcomplanate, slender plant, with concave, ovate, more or less symmetrical, plicate leaves, with recurved margins and a curled, denticulate, hyaline and recurved leaf apex. **Ecology**. It is listed in epiphytic habitat [7]. **Distribution**. The species is described as endemic of Bhutan and Nepal [7], present in evergreen forests around 3000 m. According to Wynns et al. [23], this taxon should be searched for elsewhere, as it could reasonably be expected to occur in Sikkim (Northeastern India)—which borders Bhutan in the east and Nepal in the west—and in Yunnan (China).
11. *Plagiothecium denticulatum* (Hedw.) Schimp 1851—a fairly large plant, green to yellowish green, often glossy (Figure 2); the stem prostrate to ascending, densely foliate; concave, ovate to lanceolate, asymmetrical, acute to acuminate leaves, with recurved margins (Figure 3), an often denticulate apex, and loose cell areolation (Figure 4); median cells linear-rhomboidal; alar regions well-developed, broadly decurrent, composed of large, inflated, round hyaline cells; sporophytes with inclined capsules. These features allow this species to be distinguished from others. **Ecology**. It is considered as a circumboreal species, where it is recorded in epigeic, epilithic, epixylic and epiphytic habitats; more abundant in alpine areas (e.g., [28,29,30,34,79,194,198,199,206]). **Distribution**. Apart from Eurasia, it is cited in the literature from North Africa, but considered doubtful by Ros et al. [12]; also the occurrence of this species in sub-Saharan Africa is considered doubtful by O’Shea [10]. It is also reported from North America (Canada, Greenland, USA) [7,18,25,79,207].
12. *Plagiothecium denticulatum* var. *affine* Warnst. 1906—a species with small, flat, asymmetric leaves and well-developed alar regions [7]. Warnstorf [208] stated that *P*. *denticulatum* var. *affine* has a delicate form and resembles *P*. *laetum*, and that it could be an intermediate form between these species. Intermediate features of this taxon include its delicate structure as well as straight and erect capsules (which is characteristic for all species closely related to *P*. *laetum*). Wynns [7] supports this opinion that it may be a hybrid between these two species. **Ecology**. Warnstorf [208] did not provide details about the ecological preferences of this taxon, however, he indicated that it is a delicate form growing in flat turfs. **Distribution**. So far, it is reported only from Germany (Bärwalde, between Vietnitz and Nordhausen, Königsberg) [7,208].
13. *Plagiothecium denticulatum* var. *obtusifolium* (Turner) Moore 1873—a small, subjulaceous, glossy, soft plant; leaves round, ovate or elliptical, with an obtuse apex, recurved margins and well-developed alar regions. These features distinguish this taxon from other species. *Plagiothecium denticulatum* var. *obtusifolium* was treated by some scientists as a synonym of *P*. *denticulatum* (e.g., [18,159]) or considered to be an ecotypic variety, but DNA sequence analyses indicate that *P*. *denticulatum* var. *obtusifolium* and *P*. *denticulatum* are not the same taxon [7,23]. **Ecology**. Wynns [7] reported on that *P*. *denticulatum* var. *obtusifolium* is restricted to mountains and cliffs, where it is recorded in epigeic, epilithic and epiphytic habitats. **Distribution**. Outside Eurasia, the species reported from North America (Canada, USA) [7,79].
14. *Plagiothecium enerve* (Broth.) Q. Zuo 2011—due to a rather unusual combination of features: small plant with not tumid branches, narrowly lanceolate leaves bordered by hyaline, elongate, thin-walled cells, with an extremely long, often brownish piliferous apex, this plant was first described as *Struckia enervis* [209]. However, DNA analysis [36] indicates that it belongs to the genus *Plagiothecium*. **Ecology**. This species is recorded in epilithic and epiphytic habitats [7]. **Distribution**. Until now, it is reported from Asia (China and Russia).
15. *Plagiothecium euryphyllum* (Cardot & Thér.) Z. Iwats. 1970—a medium-sized to robust, glossy plant, with flattened branches; leaves ovate to elliptical, asymmetric, slightly contorted when dry, broadly acute, more or less undulate; median leaf cell linear, narrow, thin-walled, areolation looks tight. Due to this feature combination, *P*. *euryphyllum* is similar and confused with *P*. *neckeroideum*, but this species does not have intense iridescent and concave leaves as *P*. *euryphyllum*. **Ecology**. The species is recorded in epigeic, epilithic, epixylic and epiphytic habitats [29,64,79]. **Distribution**. It is widely spread over Asia (China, Formosa, Japan, Korea and Myanmar) and Eastern Europe (Russia) [7,29,32,72,140].
16. *Plagiothecium fallax* Cardot & Thér. 1902—Cardot & Thériot [210] described this species as a robust, light green, shiny plant; leaves broadly ovate-lanceolate, asymmetrical, undulate, with a broad base and wider areolation of thin-walled cells. According to these authors, *P*. *fallax* is similar to *P*. *denticulatum sensu lato*, but it can be distinguished from this species by very small alar decurrencies. Ireland [18] and Iwatsuki [29] treated this species as a synonym or variety of *P*. *cavifolium*. **Ecology**. This species is recorded in epigeic habitat. **Distribution**. Apart from Eurasia (Russian Federation and Japan), it is also reported from North America (USA) and considered to be a typical North Pacific element [7].
17. *Plagiothecium japonicum* Sakurai 1949—described by Sakurai [211], but Iwatsuki [29] treated this species as a form of *P*. *nemorale* (*P*. *nemorale* fo. *japonicum*), later even as a synonym to this species [33]. *Plagiothecium japonicum* can be easily recognised by large, broadly ovate, often concave leaves with stiff, extended cells and, as indicated by Wynns [7], it should be treated as a separate species, despite the fact that morphologically and genetically it shows intermediate features between *P*. *nemorale* and *P*. *cavifolium*. **Ecology**. This species is recorded in epigeic and epilithic habitats. **Distribution**. Apart from Asia (Japan), it is also reported from North America (USA) and considered a North Pacific element as *P. fallax* [7].
18. *Plagiothecium laetum* Schimp. 1851—small plant, pale green to yellowish green, glossy, in loose mats; leaves asymmetrical, narrowly ovate-lanceolate, and narrowly-decurrent, gradually acuminate at apex; median leaf cells linear-rhomboidal; capsules more or less erect. Narrow alar decurrencies and sporophytes with erect capsules easily distinguish this species from closely related ones such as *P*. *curvifolium*. A recent taxonomic study of *P*. *laetum* complex allowed description of a new species, i.e., *P*. *rossicum* [26]. **Ecology**. The species recorded in epigeic, epilithic, epixylic, and epiphytic habitats (e.g., [25,28,30,66,79,171,172,195,212]). **Distribution**. Apart from Eurasia, this species is also reported from North America (Canada, Greenland, USA) [7,18,79].
19. *Plagiothecium laetum* var. *tenellum* (Schimp.) Warnst. 1906—Warnstorf [208] states that this taxon differs from *P*. *laetum* by longer, more lanceolate leaves, as well as narrow and long cells. Jedlička [202,203] characterised *P*. *laetum* var. *tenellum* as having small, narrow leaves, very short costae and narrow cells, often with propagules, and small erect capsules. **Ecology and Distribution**. Warnstorf [208] did not provide data about the ecological preferences of this taxon, therefore detailed ecological data and distribution for this taxon are not known exactly and require specific investigation. However, he indicated that var. *tenellum* plants are from all locations listed to *P*. *laetum*, which include Germany (Crossen, Lübeck, Hamburg and Altmark). The ecological data reported for these locations were: very rare in pine and deciduous forests at the bottom of trees or on forest floor, sometimes also in moors on the edge of old peat holes; often in crevices of lower and higher mountains.
20. *Plagiothecium latebricola* Wilson ex Schimp. 1851—a small, slender, bright green or yellowish green, glossy plant; leaves erect-spreading, symmetrical, narrowly ovate-lanceolate, sometimes complanate, long acuminate, at times shrunken when dry, a margin narrowly recurved, entire or denticulate near the apex; costae very short; median leaf cells linear-rhomboidal; alar regions narrowly decurrent; fusiforme gemmae often present as well as rhizoids at the apex; capsules erect. Because of its small size, colour, short costae, narrow decurrent alar regions and erect capsules, this species can be confused with *P*. *laetum*, but even leaf symmetry in the latter helps to distinguish these two species. **Ecology**. *Plagiothecium latebricola* is found in swamps, fens, marshes and recorded in epigeic, epilithic, epixylic and epiphytic habitats (e.g., [21,25,28,30,79,101,199,206,213]). **Distribution**. This species has a circumboreal distribution [7], typically found in lowland, shaded locations [21,25]. Apart from Eurasia, it is also reported from North America (Canada, USA) [18,79].
21. *Plagiothecium longisetum* Lindb. 1872—species described by Lindberg [214], synonymized with *P*. *nemorale* by Iwatsuki [29] and treated as such for about 50 years [33], recently resurrected and considered as a separate species [27]. A robust, green to yellow green, plant without metallic lustre (Figure 2); leaves asymmetric to strongly asymmetric, shrunken when dry, ovate to lanceolate; a straight, not denticulate, acute to acuminate apex; leaf cells in regular rows, long and wide, areolation loose; long and burly costae; and very long seta (to 5.5 cm). This feature combination allows us to easily distinguish this species from *P*. *nemorale* and other closely related species. **Ecology**. It is recorded in epigeic, epilithic, epixylic and epiphytic habitats [27]. **Distribution**. Apart from Eurasia, this species is also reported from North America (Canada, USA) [191].
22. *Plagiothecium neckeroideum* Schimp. 1851—a robust, green to yellowish green, strongly complanate plant; leaves domorphic, triangular, asymmetrical, ovate, undulate, concave, and a serrulate apex; median leaf cells linear-rhomboidal, narrow; alar decurrencies hyaline, thin-walled and well-developed. Apex cells are nematogenous, leaves often with differentiated apical cells, often seen as a longitudinal brown stripe at the leaf apex. **Ecology**. It is noted in epigeic, epilithic, epixylic and epiphytic habitats (e.g., [29,34,66,79]). **Distribution**. Wynns [7] reported *P*. *neckeroideum* from East and Southeastern Asia and in the Himalayas, from Europe (in the Alps). Deng-ke and Ireland [79] also gives this species from North America.
23. *Plagiothecium neckeroideum* fo. *exile* J.T. Wynns 2015—a taxon described by Wynns [23] as a small plant, with slender stems and branches, reddish stems; concave, not undulate, acuminate leaves; leaf cell areolation composed of short and narrow cells; decurrencies with enlarged, hyaline alar cells. **Ecology**. Reported from *Quercus semecarpifolia* forest, on tree trunk (epiphytic) [23]. **Distribution**. So far, the taxon is known only from Nepal.
24. *Plagiothecium neckeroideum* var. *javense* M. Fleisch. 1920—Fleischer [215] describes this taxon as a large, light green plant, with pale, symmetric, concave, undulate, long acuminate leaves, a denticulate apex, short costae, thin-walled leaf cells, enlarged in the basal area, with a vertical stripe of nematogenous cells at the apex. **Ecology**. This taxon was recorded in epigeic and epilithic habitats. **Distribution**. Apart from Southeast Asia (Indonesia, Philippines), it is also reported from Papua New Guinea [216] and from East Africa (Ethiopia) [10].
25. *Plagiothecium neckeroideum* var. *myurum* Molendo 1875—smaller than *P*. *neckeroideum* var. *javense*, other features that make it different from closely related species are that it is a julaceous plant, with strongly concave, not undulate leaves [7,217]. **Ecology**. It is a montane taxon, recorded in epigeic, epilithic, epixylic and epiphytic habitats [7]. **Distribution**. Reported from Sino-Himalayan region (Bhutan, China, India, Nepal) [23].
26. *Plagiothecium neckeroideum* var. *niitakayamae* (Toyama) Z. Iwats. 1970—a big, light green, julaceous plant, with symmetrical, plicate leaves; this variety differs from the species by more julaceous, symmetric, undulate leaves. **Ecology**. This taxon was recorded in epigeic, epilithic and epiphytic habitats [7,29,79]. **Distribution**. Recorded from East Asia (China, Japan, Taiwan) and Southeast Asia (Philippines).
27. *Plagiothecium neckeroideum* fo. *parvum* J.T. Wynns 2015—a form proposed by Wynns [7], who describes it as a small, pale green, crispate when dry, with flat or undulate, very concave, cordate, short, broad and very asymmetrical leaves, with an acuminate, acute and denticulate apex, often with rhizoids; leaf cells narrow; this form is similar to *P*. *subglaucum*, but in *P*. *neckeroideum* fo. *parvum* the leaves are broader. **Ecology**. This taxon was recorded in epigeic habitats [7]. **Distribution**. So far, it is known only from Taiwan (East Asia).
28. *Plagiothecium nemorale* (Mitt.) A. Jaeger 1878—this species has been too widely described in the last few decades, a taxonomic review of *P*. *nemorale sensu lato* indicates that it is actually three separate species: *P*. *nemorale sensu stricto*, *P*. *longisetum* and *P*. *angusticellum* [27]. A medium to large, green to dark green plant, shrunken when dry (Figure 2) and without metallic luster; leaves ovate, symmetric (Figure 3); acute to acuminate, straight; a denticulate apex; leaf cells short and wide, loose areolation, symmetric, in regular rows (Figure 4). This feature combination makes it very easy to distinguish this species from other closely related species. **Ecology**. The species is recorded in epigeic, epilithic, epixylic, and epiphytic habitats (e.g., [25,27,28,29,34,66,79,141,194,199,200,218]). **Distribution**. The species is quite common in Eurasia [27]. It is also reported by Ros et al. [12,47] from North Africa (Algeria, Tunisia) and reported from North America (Canada, USA) [27,191].
29. *Plagiothecium noricum* Molendo ex Limpr. 1897—flaccid, not undulate, very concave leaves, with expended cell aerolation, denticulate; rhizoids at the apex. **Ecology**. Wynns [7] describes *P*. *noricum* as a still little-known Alpine species, where are listed from epigeic habitat. **Distribution**. Reported from the Southern part of Central Europe (Austria) and Southeast Asia (Myanmar).
30. *Plagiothecium obtusissimum* Broth. 1921—a yellowish green to pale green, glossy plant with metallic luster; leaves ovate, rounded-obtuse at the apex, asymmetrical, slightly concave, the margin often erect at one side; leaves with suddenly differentiated alars, composed by hyaline inflated cells; median cells linear-flexuose, very narrow and long, thin-walled. Iwatsuki [29] considered *P*. *obtusissimum* to be closely related to *P*. *euryphyllum*, due to similar alar cells, leaf cells and setae. Additionally, it is easily distinguished from this species by the plant size and shape of leaf apex. DNA analysis confirms the observations about the close relationship between these species [36]. **Ecology**. *Plagiothecium obtusissimum* is recorded in epigeic, epilithic, epixylic and epiphytic habitats (e.g., [29,34]). **Distribution**. Noguchi [34] considered this species to be endemic to Japan; however, it is later reported from Russia (e.g., [32]).
31. *Plagiothecium piliferum* (Sw.) Schimp. 1851—a small, slender, pale green and glossy plant; leaves ovate, deeply concave, almost symmetrical, abruptly contracted to a piliferous, sometimes flexuose apex, with recurved margins; median leaves linear-rhomboidal, very narrow; alar regions narrowly decurrent. **Ecology**. *Plagiothecium piliferum* is recorded in epigeic, epilithic, and epixylic habitats (e.g., [25,28,30,79]). **Distribution**. Apart from Eurasia, this species is also reported from North America (Canada, Greenland, USA) in low to moderate elevations [7,21,30,79].
32. *Plagiothecium platyphyllum* Mönk. 1927—a medium-sized to robust, green, glossy plant; leaves ovate-lanceolate, asymmetrical, complanate, facid, undulate; median leafcells linear-romboidal, apical cells often bearing rhizoids; alar cells hyaline to pale green. According to Ireland [18], it is an autopolyploid of *P*. *denticulatum*, while a DNA study [219] suggested that *P*. *denticulatum* var. *obtusifolium* is a haploid of *P*. *platyphyllum*. **Ecology**. *Plagiothecium platyphyllum* is recorded in epigeic, epilithic, epixylic, and epiphytic habitats (e.g., [25,28,30,79,128,194]). **Distribution**. Apart from Eurasia, it is also reported from North America (Canada, USA) [7,23].
33. *Plagiothecium rhizophyllum* Sakuri 1932—described by Sakurai [220] as a small species, with not undulate, loose cell areolation and rhizoids at the apex. Iwatsuki [29,221] and researchers after him (e.g., [33]) consider it as a synonym of *P*. *nemorale*, but Wynns [7] treats *P*. *rhizophyllum* as a separate species. **Ecology**. It is recorded in epigeic habitat [7]. **Distribution**. So far, the species reported only from East Asia (China).
34. *Plagiothecium rossicum* Ignatov & Ignatova 2019—described on the basis of DNA analyses of the *P*. *laetum* complex by Ignatova et al. [26]. A small plant with distinctly complanate foliage; leaves asymmetrical, ovate-lanceolate, a narrowly acute to short acuminate apex, margins flat, entire or minutely denticulate at the apex; leaf cells long and very narrow; straight and erect capsules. In terms of many features, this species is similar to *P*. *laetum*, but a flat margin and strongly asymmetric leaves are very useful in distinguishing *P*. *rossicum* from this species. Many features allow this species to be distinguished also from *P*. *svalbardense*, including for example: A flat leaf margin, narrow cells, narrowly acute to short acuminate apex which characterise *P*. *rossicum*. **Ecology**. So far, this species has been recorded in epigeic, epilithic, epixylic and epiphytic habitats [26]. **Distribution**. The species in common in boreal and hemiboreal forests of Russia, one position is also given from Poland [26].
35. *Plagiothecium ruthei* Limpr. 1897—a medium-sized to large plant; leaves strongly complanate on the stem, transversely undulate when moist, sometimes shrunken when dry, flaccid, acuminate, strongly asymmetrical, on side almost straight, leaves with narrowly recurved margins and well-developed alar regions. These features distinguish this species from other closely related species. It is recognised as a separate species throughout Eurasia, despite the fact that DNA data places this plant closer to *P*. *denticulatum*, even closer than *P*. *denticulatum* var. *obtusifolium* [7,23]. **Ecology**. *Plagiothecium ruthei* is typical of wetlands species, recorded in epigeic, epixylic, and epiphytic habitats (e.g., [25,30,194]). **Distribution**. Apart from Eurasia, this species is also reported from North Africa but considered doubtful by Ros et al. [12]; and from North America (Canada, USA) [7].
36. *Plagiothecium ruthei* var. *rupincola* Limp. 1897—Limpricht [222] described this taxon as similar to *P*. *ruthei* due to the size and cell areolation, but different due to closer foliage; symmetrical leaves, lacking recurved margin. **Ecology**. Limpricht [222] described it as an epilithic, alpine taxon. **Distribution**. According to the protologue and Wynns [7], this taxon is reported from Central, Northern and Western Europe (Asutria, Czech Republic, France, Germany, Norway and Sweden).
37. *Plagiothecium subglaucum* Thwaites & Mitt. 1873—Mitten [223] described this species as a plant with ovate, flat leaves, with an acute to acuminate apex. *Plagiothecium subglaucum* is similar to and can be confused with *P*. *neckeroideum*. Both species require further in-depth research [7]. **Ecology**. It is recorded in epigeic and epiphytic habitats. **Distribution**. So far, known only from Sri Lanka (South Asia) and Myanmar (Southeast Asia).
38. *Plagiothecium succulentum* (Wilson) Lindb. 1865—a robust, yellowish green to golden green, very glossy plant, leaves symmetric, lanceolate, not shrunken when dry, with an entire apex; median leaf cells very long. *Plagiothecium succulentum* differs from *P*. *nemorale* by lanceolate leaves, longer cells and a smooth apex; and from *P*. *longisetum* by lanceolate, symmetrical leaves; from other closely related species (e.g., *P*. *angusticellum*), it is very easy to distinguish, for example, by leaf symmetries and loose cells areolation [27]. Wynns [7] considered *P*. *succulentum* as problematic and described this species as polyphyletic or intermediate between *P*. *nemorale* and *P*. *cavifolium*. *Plagiothecium succulentum* in some countries is indicated as doubtful (Table 2, Figure S4). In our opinion, the relationship between these above-mentioned species requires a detailed analysis. **Ecology**. *Plagiothecium succulentum* is recorded in epigeic, epilithic, and epiphytic habitats (e.g., [25,28,30,62,79,194]). **Distribution**. Apart from Eurasia, this species is also reported from North Africa (a single record from Tunisia) [47]; and from North America (Canada, USA) [191].
39. *Plagiothecium succulentum* fo. *propaguliferum* E. Bauer 1902—a very dark, small plant, with shrunken leaves when dry. These are the features that distinguish this form from *P*. *succulentum*. Wynns [7] commented that this taxon can be frequently found in herbaria under the name *P*. *succulentum*. **Ecology**. This taxon is recorded in epilithic and epiphytic habitats [7]. **Distribution**. Currently, *P*. *succulentum* fo. *propaguliferum* is recorded in Western, Northern and Western Europe [7], and from North America (Canada, USA) [7,191].
40. *Plagiothecium svalbardense* Frisvoll 1996—a small, growing erect plant, crispy when dry; leaves small, weakly undulate, concave, symmetrical to slightly asymmetrical, short, ovate, gradually tapered to the apex; margins narrowly recurved, entire or minutely denticulate at the apex; a subpiliferous apex; capsules straight and erect. *Plagiothecium svalbardense* is different from *P*. *laetum* by leaf shape and apex shape, the described species is also similar to *P*. *piliferum* due to its apex, but the latter has narrower leaf cells and a longer apex. The shape and arrangement of capsules is similar to *P*. *laetum* and *P*. *berggrenianum* but different from *P.* *curvifolium*. **Ecology**. Wynns [7] described *P*. *svalbardense* as an arctic species, where it is recorded in epilithic and epixylic habitats [26]. **Distribution**. Apart from Eurasia (Russia, Svalbard, Sweden), this species is also reported from North America (Greenland) [7].
41. *Plagiothecium undulatum* (Hedw.) Schimp. 1851—a large white to pale green, dull plant; leaves large, imbricate, crispate, slightly asymmetric, acute and serrulate at the apex; rhizoids occur at the leaf insertion; leaf cells with papillae on abixal surfaces. These mentioned features led Ireland [224] to create for this species (as well as *P*. *draytonii* (Sull.) E.B. Bartram) a separate genus—*Buckiella* Ireland. *Plagiothecium undulatum* is similar to *P*. *neckeroideum*, but it differs by size and colour of the plant as well as longer and broader median leaf cells. **Ecology**. *Plagiothecium undulatum* was recorded in epigeic, epilithic, and epiphytic habitats (e.g., [25,28,30,79,197]). **Distribution**. Apart from Eurasia, this species is also reported from North America (Canada, USA) [7,18,79].

## 3. Materials and Methods

### 3.1. The Area of the Checklist

**Figure 5:**
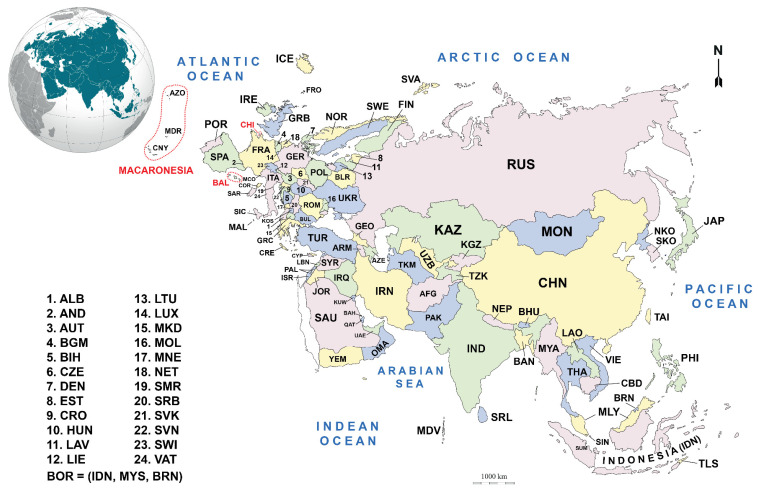
Map of the study area showing the location and outline of the surveyed countries. The abbreviations used for the countries are included separately in the main text (cf. Table 1).

The present study covers Eurasia (Figure 5), which comprises all of Europe and Asia, excluding Papua New Guinea. It also includes archipelagos of Northern Macaronesia, i.e., the Azores, Madeira and the Canary Islands but excl. the Cape Verde (Cabo Verde) archipelago. From the political and administrative perspective, Eurasia includes Europe and Asia (excl. the Sinai Peninsula, which is usually linked to Africa; being part of Egypt, though geographically it is located in Asia; anyhow *Plagiothecium* had never been reported from Sinai, see [225]). The area covered by the present checklist includes 107 countries of Europe and Asia, some well-separated islands, and archipelagos. According to the “World Geographical Scheme for Recording Plant Distributions” by Brummitt [226], it includes:

- Europe (Northern, Middle, Southwestern, Southeastern, and Eastern Europe);
- Asia-Temperate (Siberia, Russian Far East, Middle Asia, Caucasus (excl. partially recognised countries such as Abkhazia), Western Asia, the Arabian Peninsula, China, Mongolia, and Eastern Asia); and
- Asia-Tropical (Indian Subcontinent, Indo-China, Malaysia, but excl. Papuasia).

A list of Eurasian countries and islands, along with their abbreviations used throughout the text, is presented in alphabetical order in Table 1. Abbreviations of countries and regions follow TDWG geographical codes [226] with some exceptions. The resulting distribution maps of the accepted taxa are provided as Supplementary Materials (Figures S1–S4).

### 3.2. Data Collection and Presentation

This study is based primarily on information garnered from the literature available to the authors up to the end of June 2020. More than two hundred publications have been consulted and analysed, including important regional and local floras, checklists, floristic reports, various studies and revisions of the family Plagiotheciaceae and the genus *Plagiothecium* in all European and Asian biogeographic regions or countries. Information on species names, occurrence, distribution, ecology and taxonomy have been compiled from these publications, which covered almost all the work published on *Plagiothecium* over a period of more than 70 years by several authors and written in different languages. In addition, the authors’ own observations and online databases/checklists available on bryophytes of several regions/countries under consideration were used. All synonyms appearing in the collected literature were compiled for each taxon (see Appendix A).

The Eurasian regions are arranged alphabetically and a regional distribution is reported for each individual taxon. Data are presented in tabular format (Table 2) and distribution maps are provided for all reported taxa (see Figures S1–S4). Sections are arranged phylogenetically. Within sections, taxa are arranged alphabetically, first by the generic name, then by the specific epithet and infraspecific name—without any consideration of their phylogenetic relationships. Entries within each section follow the order: Taxon name, authority, publication data (pertaining to taxon first valid publication), then followed by distribution data (literature references for each country from which the taxon is reported), see Appendix B. Abbreviations of countries are printed in **boldface**. Author abbreviations follow [227].

The frequency of occurrence of Eurasian species has been presented on a five-point scale: very rare species (from 1% to 5% of all countries/islands in which a given taxon occurred), rare (6–10%), frequent (11–25%), very frequent (from 26–50%) and common (more than 50% of all countries/islands in which the taxon occurs).

### 3.3. Nomenclature and Taxonomy

The revised sectional classification of *Plagiothecium* established by Wynns [7] based on phylogenetic analysis of DNA sequence data, is adopted in the present study with a few exceptions. Consequently, the following taxa have been excluded from the present study: *P*. *handelii* Broth. and *P*. *paleaceum* (Mitt.) A. Jaeger, which after Wynns and Schröck [37] were sanctioned as *Ortholimnobium handelii* (Broth.) C. Schröck & J.T. Wynns and *O.* *paleaceum* (Mitt.) C. Schröck & J.T. Wynns, respectively. Whereas taxa considered in this study and not included in [7] are: *P*. *rossicum* Ignatov & Ignatova, which was established by Ignatova et al. [26] based on phylogenetic analysis of the *P*. *laetum* complex; as well as *P*. *angusticellum* G.J. Wolski & P. Nowicka-Krawczyk and *P*. *longisetum* Lindb., which were erected and resurrected, respectively, based on application of polyphasic approach to investigate the *P*. *nemorale sensu lato* [27].

### 3.4. Conspectus of Classification of Plagiothecium

All species are classified into the sections proposed by Wynns [7] and Wynns et al. [23], while for species that were described/accepted after 2015, we suggested that they can be provisionally classified under *Plagiothecium* sect. *Leptophyllum* (for *P*. *rossicum*) and *Plagiothecium* sect. *Orthophyllum* (for *P*. *angusticellum* and *P*. *longisetum*) and so this was adopted and included below (based on the literature cited in the two sections above).

All the following sections are represented in Eurasia.

*Note.* Included taxa listed here are as provided in [7,23,26,27,37], infraspecific taxa are not included.

**Plagiotheciaceae** M.Fleisch., Nova Guinea 8: 748. 1912;
Hypnaceae subfam. Plagiothecioideae Brotherus *in* Engler and Prantl, Nat. Pflan-
zenfam. 1(3): 1021, 1078. 1908, “Plagiothecieae”. Type. *Plagiothecium* Schimper.

**Plagiothecium** Schimper in Bruch, Schimper and Gümbel, Bryol. Eur. 5:179. 1851 ≡
*Stereodon* (Bridel) Mitten sect. *Plagiothecium* (Schimper) Mitten, J. Linn. Soc., Bot. 4:
88. 1859. Type. *Plagiothecium denticulatum* (Hedwig) Schimper in Bruch, Schimper and Gümbel, Bryol. Eur.

1. ***Plagiothecium*** Schimp. sect. ***Plagiothecium*** ≡ *Plagiothecium* sect. *Falciphyllum* Jedl., nom. illeg. Spisy Vydá. Přír. Fak. Masarykovy Univ. 308: 23. 1948. = *Plagiothecium* sect. *Rostriphyllum* Jedl. Spisy Vydá. Přír. Fak. Masarykovy Univ. 308: 32. 1948.
  This section consists of 12 species: *P. brasiliense* (Hampe) A. Jaeger, *P. cochleatum* Dixon, *P. conostegium* Herzog, *P. denticulatum* (Hedw.) Schimp, *P. lamprostachys* (Hampe) A. Jaeger, *P. berggrenianum* Frisvoll, *P. membranosulum* Müll. Hal., *P. nitens* Dixon, *P. ovalifolium* Cardot, *P. platyphyllum* Mönk., *P. ruthei* Limpr., *P. selaginelloides* Müll. Hal.
2. ***Plagiothecium*** sect. ***Orthophyllum*** Jedl. Spisy Vydá. Přír. Fak. Masarykovy Univ. 308: 35. 1948.
  This section consists of 7 species: *P*. *angusticellum* G.J. Wolski & P. Nowicka-Krawczyk, *P. cavifolium* (Brid.) Z. Iwats., *P. japonicum* Sakurai, *P*. *longisetum* Lindb., *P. nemorale* (Mitt.) A. Jaeger, *P. rhizophyllum* Sakurai, *P. succulentum* (Wilson) Lindb.
3. ***Plagiothecium*** sect. ***Leptophyllum*** Jedl. Spisy Vydá. Přír. Fak. Masarykovy Univ. 308: 23. 1948. = *Plagiothecium* sect. *Philoscia* (Berk.) Ochyra. Biodiversity of Poland 3: 177. 2003. ≡ *Philoscia* Berk. Handbook of British Mosses 49, 146. 1863.
  This section consists of 12 species: *P. andinum* (Hampe) A. Jaeger, *P. curvifolium* Schlieph. ex Limpr., *P. funale* J.T. Wynns, *P. laetum* Schimp., *P. latebricola* Wilson ex Schimp., *P. lucidum* (Hook. f. and Wilson) Paris, *P. mollicaule* R.S. Williams, *P. pacificum* J.T. Wynns, *P. rhizolucidum* J.T. Wynns, *P*. *rossicum* Ignatov & Ignatova, *P. svalbardense* Frisvoll.
4. ***Plagiothecium*** sect. ***Pseudo-Neckera*** (Kindb.) J.T. Wynns. Cladistics 34: 469–501. 2018. ≡ *Plagiothecium* subgen. *Pseudo-Neckera* Kindb. European and North American Bryineae (Mosses) 1: 69. 1897.
  This section consists of 4 species: *P. decoratum* J.T. Wynns, *P. neckeroideum* Schimp., *P. noricum* Molendo ex Limpr., *P. subglaucum* Thwaites & Mitt.
5. ***Plagiothecium*** sect. ***Lycambium*** Jedl. 1948. Spisy Vydá. Přír. Fak. Masarykovy Univ. 308: 10. 1948. ≡ *Buckiella* Ireland. Novon 11(1): 55. 2001.
  This section consists of 3 species: *P. draytonii* (Sull.) E.B. Bartram, *P. fallax* Cardot & Thér., *P. undulatum* (Hedw.) Schimp.
6. ***Plagiothecium*** sect. ***Saviczia*** (Abramova & I.I. Abramov) Z. Iwats. J. Hattori Bot. Lab. 33: 341. 1970. ≡ *Saviczia* Abramova & I.I. Abramov. Novosti Sist. Nizsh.Rast. 1966: 298. 1966.
  This section consists of 2 species: *P. euryphyllum* (Cardot & Thér.) Z. Iwats., *P. obtusissimum* Broth.
7. ***Plagiothecium*** sect. ***Struckia*** (Müll. Hal.) J.T. Wynns. Cladistics 34: 469–501. 2018. ≡ *Struckia* Müll. Hal. Arch. Vereins Freunde Naturgesch. Mecklenburg 47: 129. 1893.
  This section consists of 2 species: *P. argentatum* (Mitt.) Q. Zuo, *P. enerve* (Broth.) Q. Zuo.
8. ***Plagiothecium*** sect. ***Rectithecium*** (Hedenäs & Huttunen) J.T. Wynns. Cladistics 34: 469–501. 2018. ≡ *Rectithecium* Hedenäs & Huttunen. Bot. J. Linn. Soc. 171(2): 344. 2013.
  This section consists of one species: *P. piliferum* (Sw.) Schimp.

Note: All sections proposed by Wynns [7] and Wynns et al. [23] are represented in Eurasia except *Plagiothecium* sect. *Ortholimnobium*. It should be noted, however, that this section includes the two species *P. handelii* and *P. paleaceum*, which are not considered in the present study in light of the new evidence published by Wynns and Schröck [37], as explained above.

## Figures and Tables

**Table 1:** Abbreviation of countries/regions treated in the checklist, with sources of the regional information.

No.	Country/Region	Abbreviation	Main Source(s) of Information
1.	Afghanistan	AFG	[43,44,45,46]
2.	Albania	ALB	[9,47,48,49,50]
3.	Andorra	AND	[9,47,48,51,52]
4.	Armenia	ARM	[32,53]
5.	Austria	AUT	[7,9,26,27,48,54]
6.	Azerbaijan	AZE	[32]
7.	Azores (Portugal)	AZO	[9,27,47,48,55,56,57]
8.	Bahrain	BAH	[43,45]
9.	Balearic Islands (Spain)	BAL	[47,48,58]
10.	Bangladesh	BAN	[59,60]
11.	Belarus	BLR	[9,32,48,61]
12.	Belgium	BGM	[7,9,27,48,62]
13.	Bhutan	BHU	[7,63,64]
14	Borneo (Indonesia, Malaysia, Brunei)	BOR	[65,66]
15.	Bosnia and Herzegovina	BIH	[9,26,47,48,49]
16.	Brunei Darussalam	BRN	[67]
17.	Bulgaria	BUL	[9,47,48,49,68]
18.	Cambodia	CBD	[69,70,71,72,73]
19.	Canary Islands (Spain)	CNY	[9,47,48,74,75]
20.	Channel Islands (United Kingdom)	CHI	[9,48]
21.	China	CHN	[7,27,36,64,66,76,77,78,79,80]
22.	Corsica (France)	COR	[9,47,48,62]
23.	Crete (Greece)	CRE	[47,48,81]
24.	Croatia	CRO	[9,47,48,49,82,83]
25.	Cyprus	CYP	[43,47,48]
26.	Czech Republic	CZE	[7,9,26,27,48,84]
27.	Democratic People’s Republic of Korea (North Korea)	NKO	[7,64,66,78,85,86]
28.	Denmark	DEN	[7,9,27,30,48,87]
29.	Estonia	EST	[9,27,32,48,88]
30.	Faroe Islands (Denmark)	FRO	[7,9,48,89,90]
31.	Finland	FIN	[7,9,26,27,28,48,91,92,93]
32.	France	FRA	[7,9,27,47,48,78,94]
33.	Germany	GER	[7,9,26,27,48,95,96,97,98]
34.	Greece	GRC	[9,47,48,49]
35.	Hungary	HUN	[7,9,26,27,48,99,100,101]
36.	Iceland	ICE	[7,9,48,102]
37.	India	IND	[7,27,64,78,103,104]
38.	Indonesia	IDN	[7,105]
39.	Iraq	IRQ	[43,45,106,107,108]
40.	Ireland	IRE	[9,25,48,109]
41.	Islamic Republic of Iran	IRN	[7,27,43,45,110,111]
42.	Israel	ISR	[43,45,47,112,113]
43.	Italy	ITA	[7,9,27,47,48,114,115,116,117]
44.	Japan	JAP	[7,27,29,33,34,64,66,78,118,119]
45.	Jordan	JOR	[43,45,47]
46.	Kazakhstan	KAZ	[32,48]
47.	Kosovo	KOS	[9,48,120]
48.	Kuwait	KUW	[43,45,121,122]
49.	Kyrgyzstan	KGZ	[32]
50.	Lao People’s Democratic Republic	LAO	[72,78,123]
51.	Latvia	LAV	[7,9,27,32,48,124,125]
52.	Lebanon	LBN	[43,45,47]
53.	Liechtenstein	LIE	[9,48,126]
54.	Lithuania	LTU	[9,27,32,48,127,128]
55.	Luxembourg	LUX	[9,48,129,130]
56.	Madeira (Portugal)	MDR	[9,27,47,48,51,131,132]
57.	Malaysia	MLY	[133]
58.	Maldives	MDV	[134,135]
59.	Malta	MAL	[47,48,136,137]
60.	Monaco	MCO	[48]
61.	Mongolia	MON	[138,139]
62.	Montenegro	MNE	[9,47,48,49]
63.	Myanmar (Burma)	MYA	[72,140]
64.	Nepal	NEP	[7,27,64,141,142]
65.	Netherlands	NET	[7,9,48,143,144]
66.	Norway	NOR	[7,9,26,27,48]
67.	North Macedonia (formerly Macedonia)	MKD	[9,47,48,49,145,146]
68.	Oman	OMA	[43,45,122]
69.	Pakistan	PAK	[147,148,149,150,151]
70.	Palestine	PAL	No information available
71.	Philippines	PHI	[7,66,152,153]
72.	Poland	POL	[7,9,26,27,48,154]
73.	Portugal	POR	[9,27,47,48,51,155,156]
74.	Qatar	QAT	[43,45,157]
75.	Republic of Korea (South Korea)	SKO	[7,64,66,78,86]
76.	Republic of Moldova	MOL	[9,32,48]
77.	Romania	ROM	[9,48,49,158]
78.	Russia Federation	RUS	[7,9,26,27,32,48,64,78,159,160,161,162]
79.	San Marino (Italy)	SMR	[9,48]
80.	Sardinia (Italy)	SAR	[9,47,48]
81.	Saudi Arabia	SAU	[43,45,122]
82.	Serbia	SRB	[9,47,48,49]
83.	Sicily (Italy)	SIC	[9,47,48]
84.	Singapore	SIN	[133]
85.	Slovakia	SVK	[7,9,27,48,163,164]
86.	Slovenia	SVN	[9,47,48,49,165,166]
87.	South Georgia	GEO	[27,32]
88.	Spain	SPA	[9,27,47,48,51,167]
89.	Sri Lanka	SRL	[7,168,169,170]
90.	Sumatra (Indonesia)	SUM	[66,105]
91.	Svalbard (Norway)	SVA	[7,9,26,48,171,172,173]
92.	Sweden	SWE	[7,9,26,27,48,174,175]
93.	Switzerland	SWI	[7,9,27,48,176,177]
94.	Syrian Arab Republic	SYR	[43,45,47]
95.	Taiwan	TAI	[7,66,77,78,178]
96.	Tajikistan	TZK	[32]
97.	Thailand	THA	[66,72,179]
98.	Timor-Leste	TLS	No information available
99.	Turkey	TUR	[7,9,27,43,45,47,48,49,180,181,182]
100	Turkmenistan	TKM	[32]
101	Ukraine	UKR	[9,26,32,48,183,184,185]
102	United Arab Emirates	UAE	[43,45,122]
103.	United Kingdom	GRB	[7,9,25,27,48,109]
104.	Uzbekistan	UZB	[32]
105.	Vatican City	VAT	[48,186,187]
106.	Vietnam	VIE	[72,123,188,189]
107.	Yemen	YEM	[43,45,122,190]

**Table 2:** List of *Plagiothecium* taxa recorded in Eurasia, showing distribution across the individual countries/islands. (1) *P*. *angusticellum*, (2) *P*. *argentatum*, (3) *P*. *berggrenianum*, (4) *P*. *cavifolium*, (5) *P*. *cavifolium* var. *orthocladium*, (6) *P*. *cochleatum*, (7) *P*. *conostegium*, (8) *P*. *curvifolium*, (9) *P*. *curvifolium* fo. *julaceum*, (10) *P*. *decoratum*, (11) *P*. *denticulatum*, (12) *P*. *denticulatum* var. *affine*, (13) *P*. *denticulatum* var. *obtusifolium*, (14) *P*. *enerve*, (15) *P*. *euryphyllum*, (16) *P*. *fallax*, (17) *P*. *japonicum*, (18) *P*. *laetum*, (19) *P*. *laetum* var. *tenellum*, (20) *P*. *latebricola*, (21) *P*. *longisetum*, (22) *P*. *neckeroideum*, (23) *P*. *neckeroideum* fo. *exile*, (24) *P*. *neckeroideum* var. *javense*, (25) *P*. *neckeroideum* var. *myurum*, (26) *P*. *neckeroideum* var. *niitakayamae*, (27) *P*. *neckeroideum* fo. *parvum*, (28) *P*. *nemorale*, (29) *P*. *noricum*, (30) *P*. *obtusissimum*, (31) *P*. *piliferum*, (32) *P*. *platyphyllum*, (33) *P*. *rhizophyllum*, (34) *P*. *rossicum*, (35) *P*. *ruthei*, (36) *P*. *ruthei* var. *rupincola*, (37) *P*. *subglaucum*, (38) *P*. *succulentum*, (39) *P*. *succulentum* fo. *propaguliferum*, (40) *P*. *svalbardense*, (41) *P*. *undulatum*. Note. • = occurrence of species confirmed; **? =** doubtful occurrence; Σ_1_ = total number of *Plagiothecium* taxa recorded per country/island; Σ_2_ = total number of records for each taxon in Eurasia.

Countries/Islands	*Plagiothecium* Taxa Recorded in Eurasia																																									Σ_1_
	1	2	3	4	5	6	7	8	9	10	11	12	13	14	15	16	17	18	19	20	21	22	23	24	25	26	27	28	29	30	31	32	33	34	35	36	37	38	39	40	41	
**Afghanistan**																																										**–**
**Albania**				•				•			•							•										•										•				**6**
**Andorra**				•							•							•										•			•	•						•				**7**
**Armenia**											•																	•														**2**
**Austri** **a**				•				•			•		•					•		•	•	•						•	•			•			•	•		•	•		•	**16**
**Azerbaijan**				•							•							•										•													•	**5**
**Azores**																					•							•										?				**3**
**Bahrain**																																										**–**
**Balearic Islands**																																										**–**
**Bangladesh**											•																															**1**
**Belarus**				•							•							•		•								•							•			?			•	**8**
**Belgium**				•				•			•							•	•	•	•							•										•			•	**10**
**Bhutan**				•						•					•							•			•			•														**6**
**Bosnia and Herzegovina**				•				•			•							•										•										•			•	**7**
**Brunei Darussalam**																																										**–**
**Bulgaria**				•				•			•		•					•										•				•			•			•			•	**10**
**Cambodia**																																										**–**
**Canary Islands**																												•										•				**2**
**Channel Islands**								•			•																	•										•			•	**5**
**China**		•		•				•			•		•	•	•			•		•	•	•			•	•		•			•	•	•		•			•			•	**20**
**Corsica**				•				•			•																	•			•	•						•				**7**
**Crete**																																										**–**
**Croatia**				•				•			•							•										•				•									•	**7**
**Cyprus**																																										**–**
**Czech Republic**	•			•				•			•		•					•		•		•						•				•			•	•		•	•		•	**15**
**Democratic People’s Republic of Korea**				•							•				•			•				•						•			•	•						•				**9**
**Denmark**				•				•			•							•		•	•							•			•	•			•			•	•		•	**13**
**Estonia**	•			•				•			•							•		•	•							•							•			•			•	**11**
**Faroe Islands**				•	•						•																	•										•			•	**6**
**Finland**				•	•			•			•		•					•		•	•							•			•	•			•			•			•	**14**
**France**				•				•			•		•					•		•	•							•			•	•			•	•		•			•	**14**
**Germany**				•				•			•	•	•					•		•	•	•						•				•			•	•		•	•		•	**16**
**Greece**				•				•			•							•										•				•						•				**7**
**Hungary**	•			•				•			•		•					•		•								•				•			•			•			•	**12**
**Iceland**				•							•		•																									•				**4**
**India**		•		•		•					•				•						•	•			•			•														**9**
**Indonesia**																						•		•																		**2**
**Iraq**											•																															**1**
**Ireland**				•				•			•		•					•		•								•			•	•			•			•			•	**12**
**Islamic Republic of Iran**				•							•		•					•			•							•				•						•			•	**9**
**Israel**																																										**–**
**Italy**				•				•			•		•					•		•								•			•	•			•			•			•	**12**
**Japan**				•			•	•			•		•		•	•	•	•		•	•	•				•		•		•	•	•			•							**18**
**Jordan**																																										**–**
**Kazakhstan**				•							•							•																								**3**
**Kosovo**								•			•		•					•										•				•						•				**7**
**Kuwait**																																										**–**
**Kyrgyzstan**											•							•		•																						**3**
**Lao People’s Democratic Republic**				•																																						**1**
**Latvia**	•			•				•			•							•		•								•			•				•			•	•		•	**12**
**Lebanon**																																										**–**
**Liechtenstein**				•				•			•							•										•							•						•	**7**
**Lithuania**	•			•				•			•							•		•								•				•			•			•			•	**11**
**Luxembourg**				•				•			•		•					•		•								•				•			•			•			•	**11**
**Madeira**											•							•			•							•										•				**5**
**Malaysia**																						•																				**1**
**Maldives**																																										**–**
**Malta**																																										**–**
**Monaco**																																										**–**
**Mongolia**				•							•							•																								**3**
**Montenegro**				•				•			•		•					•										•				•			•			•			•	**10**
**Myanmar**															•													•	•								•					**4**
**Nepal**		•		•						•	•		•								•	•	•		•			•														**10**
**Netherlands**				•				•			•		•					•		•								•							•			•			•	**10**
**North Macedonia**				•				•			•																	•				•						•				**6**
**Norway**				•				•			•							•		•	•							•			•	•				•		•		•	•	**13**
**Oman**																																										**–**
**Pakistan**				•							•									•								•														**4**
**Palestine**																																										**–**
**Philippines**																						•		•		•		•														**4**
**Poland**	•			•				•			•		•					•		•	•							•				•		•	•			•			•	**14**
**Portugal**				•							•							•		•								•			•	•						•			•	**9**
**Qatar**																																										**–**
**Republic of Korea**				•							•				•			•				•						•			•	•						•				**9**
**Republic of Moldova**				•																																						**1**
**Romania**				•				•			•							•		•		•						•			•	•			•			•			•	**12**
**Russian Federation**			•	•				•			•		•	•	•	•		•		•		•						•		•	•	•		•	•			•		•	•	**20**
**San Marino**																																										**–**
**Sardinia**											•																	•			•											**3**
**Saudi Arabia**																																										**–**
**Serbia**				•				•			•							•		•								•				•						•			•	**9**
**Sicily**				•							•																	•													•	**4**
**Singapore**																						?																				**1**
**Slovakia**				•				•			•							•		•								•				•			•			•			•	**10**
**Slovenia**				•				•			•		•					•				•						•			•	•						•			•	**11**
**South Georgia**				•							•							•		•	•							•				•						•				**8**
**Spain**				•				•			•		•					•			•							•			•	•			•			•			•	**12**
**Sri Lanka**																				•																	•					**2**
**Sumatra**																						•		•																		**2**
**Svalbard**			•								•																													•		**3**
**Sweden**				•	•			•			•		•					•		•	•							•			•	•			•	•		•		•	•	**16**
**Switzerland**				•				•	•		•		•					•		•	•	•						•			•	•			•			•			•	**15**
**Syrian Arab Republic**																																										**–**
**Taiwan**				•											•			•				•				•	•	•														**7**
**Tajikistan**											•																															**1**
**Thailand**																						•																				**1**
**Timor-Leste**																																										**–**
**Turkey**				•				•			•		•					•		•	•							•			•	•						•			•	**12**
**Turkmenistan**																																										**–**
**Ukraine**				•				•			•		•					•		•		•						•			•	•			•			•			•	**13**
**United Arab Emirates**																																										**–**
**United Kingdom**				•				•			•		•					•		•	•							•			•	•			•			•			•	**13**
**Uzbekistan**																	-																									**–**
**Vatican City State**																																										**–**
**Vietnam**															•													•														**2**
**Yemen**																																										**–**
**Σ_2_**	**6**	**3**	**2**	**59**	**3**	**1**	**1**	**41**	**1**	**2**	**65**	**1**	**26**	**2**	**10**	**2**	**1**	**51**	**1**	**34**	**22**	**22**	**1**	**3**	**4**	**4**	**1**	**64**	**2**	**2**	**24**	**39**	**1**	**2**	**29**	**6**	**2**	**50**	**5**	**4**	**41**	

## Data Availability

No new data were created or analysed in this study. Data sharing is not applicable to this article.

## Supplementary Materials

The following are available online at https://www.mdpi.com/article/10.3390/plants10050868/s1, Figure S1: Maps showing the distribution of *Plagiothecium* taxa in Eurasia per country/region. A. The distribution of *P*. *argentatum*, *P*. *berggrenianum*, *P*. *cochleatum*, *P*. *conostegium*, *P*. *decoratum*, *P*. *enerve*, *P*. *euryphyllum*, *P*. *fallax*, *P*. *japonicum*, *P*. *noricum*, *P*. *obtusissimum*, *P*. *rhizophyllum*, *P*. *rossicum*, *P*. *subglaucum* and *P*. *svalbardense*. *B.* The distribution of *P*. *cavifolium* and *P*. *cavifolium* var. *orthocladium*, Figure S2: Maps showing the distribution of *Plagiothecium* taxa per country/region. A. The distribution of *P*. *curvifolium*, *P*. *curvifolium* fo. *julaceum* and *P*. *undulatum*. B. The distribution of *P*. *denticulatum*, *P*. *denticulatum* var. *affine* and *P*. *denticulatum* var. *obtusifolium*, Figure S3: Maps showing the distribution of *Plagiothecium* taxa per country/region. A. The distribution of *P*. *laetum*, *P*. *laetum* var. *tenellum* and *P*. *latebricola*. B. The distribution of *P*. *neckeroideum*, *P*. *neckeroideum* var. *javense*, *P*. *neckeroideum* var. *myurum*, *P*. *neckeroideum* var. *niitakayamae*, *P*. *neckeroideum* fo. *exile* and *P*. *neckeroideum* fo. *parvum*, Figure S4: Maps showing the distribution of *Plagiothecium* taxa per country/region. A. The distribution of *P*. *angusticellum*, *P*. *longisetum*, *P*. *nemorale*, *P*. *piliferum* and *P*. *platyphyllum.* B. The distribution of *P*. *ruthei*, *P*. *ruthei* var. *rupicola*, *P*. *succulentum* and *P*. *succulentum* fo. *p**ropaguliferum*.

## Appendix A

List of synonyms and names used in the studied area literature, with reference to the currently accepted names (in **bold**).

*Buckiella undulata* (Hedw.) Ireland = ***Plagiothecium undulatum***
*Cephalocladium enerve* (Broth.) Abramova & I. I. Abramov = ***Plagiothecium enerve***
*Fissidens denticulatus* Baumg. = ***Plagiothecium denticulatum***
*Fobronia enervis* Broth. = ***Plagiothecium enerve***
*Hypnum argentatum* Mitt. = ***Plagiothecium argentatum***
*Hypnum cavifolius* Brid. = ***Plagiothecium cavifolium***
*Hypnum denticulatum auct. non* Hedw. = ***Plagiothecium cavifolium***
*Hypnum denticulatum* L. ex Hedw. = ***Plagiothecium denticulatum***
*Hypnum denticulatum* var. *densum* (Schimp.) Lesq. & James = ***Plagiothecium denticulatum***
*Hypnum denticulatum* var. *donnianum* (Sm.) Hook. = ***Plagiothecium denticulatum***
*Hypnum denticulatum* var. *laetum* (Schimp.) Lindb. = ***Plagiothecium laetum***
*Hypnum denticulatum* var. *majus* Boulay = ***Plagiothecium denticulatum***
*Hypnum denticulatum* var. *obtusifolium* Turner = ***Plagiothecium denticulatum*** var. ***obtusifolium***
*Hypnum denticulatum* var. *piliferum* (Hartm.) Wahlenb. = ***Plagiothecium piliferum***
*Hypnum denticulatum* var. *sublaetum* Lind. = ***Plagiothecium laetum***
*Hypnum denticulatum* var. *succulentum* Wilson = ***Plagiothecium succulentum***
*Hypnum denticulatum* var. *tenellum* (Schimp.) Husn. = ***Plagiothecium laetum***
*Hypnum lamprostachys* Hamp. = ***Plagiothecium denticulatum***
*Hypnum letabricola* (Schimp.) Hobk. = ***Plagiothecium latebricola***
*Hypnum obtusifolium* (Turner) Brid. = ***Plagiothecium denticulatum*** var. ***obtusifolium***
*Hypnum orthocarpum* Aongstr. = ***Plagiothecium piliferum***
*Hypnum roeseanum* Hampe = ***Plagiothecium curvifolium***
*Hypnum silvaticum* Hedw. ex. Lindb. *nom. inval. in synon. err. orthogr. pro. Hypnum*
*sylvaticum* Brid. = ***Plagiothecium denticulatum***
*Hypnum succulentum* Wilson = ***Plagiothecium succulentum***
*Hypnum sullivantiae* Sull. & Lesq. = ***Plagiothecium cavifolium***
*Hypnum sylvaticum auct. non* Brid. = ***Plagiothecium nemorale***
*Hypnum sylvaticum* Brid. = ***Plagiothecium denticulatum***
*Hypnum trichophorum* Spruce = ***Plagiothecium piliferum***
*Hypnum trichophorum* var. *brevipile* (Schimp.) Lindb. = ***Plagiothecium piliferum***
*Hypnum undulatum* Hedw. = ***Plagiothecium undulatum***
*Isopterygiopsis piliferum* (Hartm.) Loeske = ***Plagiothecium piliferum***
*Isopterygium euryphyllum* Cardot & Thér. = ***Plagiothecium eurphyllum***
*Isopterygium latebricola* (Schimp.) Delogne = ***Plagiothecium latebricola***
*Isopterygium piliferum* (Sw.) Loeske = ***Plagiothecium piliferum***
*Leskea laeta* (Schimp.) Bergger. = ***Plagiothecium laetum***
*Leskea latebricola* (Schimp.) Wilson = ***Plagiothecium latebricola***
*Leskea pilifera* Sw. ex. Hartm. = ***Plagiothecium piliferum***
*Plagiothcium nemorale* fo. *japonicum* (Sakurai) Z. Iwats. ≡ ***Plagiothecium japonicum***
*Plagiotheciella latebricola* (Schimp.) M. Fleisch. = ***Plagiothecium latebricola***
*Plagiotheciella pilifera* (Sw.) M. Fleisch. = ***Plagiothecium piliferum***
*Plagiothecium annotinum* Stirt. ex Dixon *nom. inval.* = ***Plagiothecium denticulatum*** var. ***obtusifolium***
*Plagiothecium apiculatum* Sakurai = ***Plagiothecium cavifolium***
*Plagiothecium auritum* (Kern.) Jedl. = ***Plagiothecium ruthei***
*Plagiothecium cavifolium* fo. *otii* (Sakurai) Z. Iwats. = ***Plagiothecium cavifolium***
*Plagiothecium cavifolium* var. *fallax* (Cardot & Thér.) Z. Iwats. = ***Plagiothecium fallax***
*Plagiothecium chuzensii* Iisiba ex Sakurai = ***Plagiothecium platyphyllum***
*Plagiothecium cochlearifolimus* Dixon = ***Plagiothcium cavifolium***
*Plagiothecium crudum* Sakurai = ***Plagiothcium cavifolium***
*Plagiothecium denticulatum* fo. *laticuspis* F. Koppe = ***Plagiothecium denticulatum*** var. ***obtusifolium***
*Plagiothecium denticulatum* subsp. *aptychus* Spruce = ***Plagiothecium curvifolium***
*Plagiothecium denticulatum* subsp. *donnianum* (Sm.) Giacom. = ***Plagiothecium*** ***denticulatum***
*Plagiothecium denticulatum* subsp. *laetum* (Schimp.) Kindb. = ***Plagiothecium laetum***
*Plagiothecium denticulatum* subsp. *ruthei* (Limpr.) Kindb. = ***Plagiothecium ruthei***
*Plagiothecium denticulatum* subsp. *sulcatum* Spruc. = ***Plagiothecium denticulatum***
*Plagiothecium denticulatum* var. *aptychus* Lees in Dix. = ***Plagiothecium curvifolium***
*Plagiothecium denticulatum* var. *cryptarum* Renauld & Hérib. = ***Plagiothecium cavifo-*** ***lium***
*Plagiothecium denticulatum* var. *curvifolium* (Limpr.) Meylan = ***Plagiothecium curvi-*** ***folium***
*Plagiothecium denticulatum* var. *donnianum* (Sm.) Lindb. ex Weim. = ***Plagiothecium*** ***denticulatum***
*Plagiothecium denticulatum* var. *donnii* Lindb. = ***Plagiothecium denticulatum*** var. ***obtusifolium***
*Plagiothecium denticulatum* var. *eciliatum* Pfeff. = ***Plagiothecium laetum***
*Plagiothecium denticulatum* var. *gravetii* (Piré) Husn. = ***Plagiothecium laetum***
*Plagiothecium denticulatum* var. *imbricatum* (Boulay) Meyl. = ***Plagiothecium denticu-*** ***latum***
*Plagiothecium denticulatum* var. *laetum* (Schimp.) Lindb. = ***Plagiothecium laetum***
*Plagiothecium denticulatum* var. *laxum* Schimp. = ***Plagiothecium denticulatum***
*Plagiothecium denticulatum* var. *majus* (Boulay) Delogne = ***Plagiothecium denticulatum***
*Plagiothecium denticulatum* var. *microcarpum* Renauld & Cardot = ***Plagiothecium*** ***curvifolium***
*Plagiothecium denticulatum* var. *myurum* Schimp. = ***Plagiothecium cavifolium***
*Plagiothecium denticulatum* var. *nervosum* (Renauld) Mém. = ***Plagiothecium*** ***platyphyllum***
*Plagiothecium denticulatum* var. *orthocladium* (Schimp.) Hérib. = ***Plagiothecium cavifolium*** var. ***orthocladium***
*Plagiothecium denticulatum* var. *orthocladum* Warnst. = ***Plagiothecium denticulatum***
*Plagiothecium denticulatum* var. *phyllorhizans* Schiffn. = ***Plagiothecium denticulatum***
*Plagiothecium denticulatum* var. *podperae Jedl. = **Plagiothecium cavifolium***
*Plagiothecium denticulatum* var. *propaguliferum* (R. Ruthe ex Limpr.) Warnst. = ***Plagi-*** ***othecium denticulatum***
*Plagiothecium denticulatum* var. *recurvum* Warnst. = ***Plagiothecium curvifolium***
*Plagiothecium denticulatum* var. *roeseanum* (Schimp.) Hérib. = ***Plagio-*** ***thecium cavifolium***
*Plagiothecium denticulatum* var. *ruthei* (Limpr.) Riehm. = ***Plagiothecium ruthei***
*Plagiothecium denticulatum* var. *secundum* Lindb. = ***Plagiothecium laetum***
*Plagiothecium denticulatum* var. *sublaetum* (Lindb.) Breidl. = ***Plagiothecium laetum***
*Plagiothecium denticulatum* var. *succulentum* (Wilson) Dixon = ***Plagiothecium succu-*** ***lentum***
*Plagiothecium denticulatum* var. *sullivantiae* (Sull.) Dix. = ***Plagiothecium cavifolium***
*Plagiothecium denticulatum* var. *tenellum* Schimp. = ***Plagiothecium laetum*** var. ***tenellum***
*Plagiothecium denticulatum* var. *undulatum* R. Ruthe ≡ ***Plagiothecium ruthei***
*Plagiothecium dimorphophyllum* Sakurai = ***Plagiothecium euryphyllum***
*Plagiothecium doii* Sakurai = ***Plagiothecium euryphllum***
*Plagiothecium donnianum* (Sm.) Mitt. = ***Plagiothecium denticulatum*** var. ***obtusifolium***
*Plagiothecium erectum* Broth. = ***Plagiothecium latebricola***
*Plagiothecium euryphyllum* var. *brevirameum* (Cardot.) Z. Iwats. = ***Plagiothecium*** ***euryphyllum***
*Plagiothecium fujiyamae* Sakurai = ***Plagiothecium cavifolium***
*Plagiothecium gravetii* Piré = ***Plagiothecium laetum***
*Plagiothecium hakusanense* Sakurai = ***Plagiothecium euryphyllum***
*Plagiothecium hattorii* Sakurai = ***Plagiothecium euryphyllum***
*Plagiothecium ikedamii* Sakurai = ***Plagiothecium cavifolium***
*Plagiothecium insigne* Cardot = ***Plagiothecium fallax***
*Plagiothecium kanedae* Sakurai = ***Plagiothecium euryphyllum***
*Plagiothecium laetum* fo. *julaceum* Jedl. = ***Plagiothecium curvifolium***
*Plagiothecium laetum* fo. *tenellum* (Schimp.) Mönk. = ***Plagiothecium laetum*** var. ***tenellum***
*Plagiothecium laetum* subsp. *curvifolium* (Schlieph. ex Limpr.) Szafran = ***Plagiothecium*** ***curvifolium***
*Plagiothecium laetum* subsp. *succulentum* fo. *longifolium* (Mönk.) Jedl. = ***Plagi-*** ***othecium succulentum***
*Plagiothecium laetum* var. *curvifolium* (Limpr.) Mastracci & M. Sauer = ***Plagiothecium*** ***curvifolium***
*Plagiothecium laetum* var. *densum* (Schimp.) Warnst. = ***Plagiothecium laetum***
*Plagiothecium laetum* var. *secundum* (Lindb.) Frisvoll et al. = ***Plagiothecium curvifo-*** ***lium***
*Plagiothecium laetum* var. *sublaetum* (Breidl.) Warnst. = ***Plagiothecium laetum***
*Plagiothecium laetum* var. *tenellum* (Schimp.) Warnst. = ***Plagiothecium laetum***
*Plagiothecium latifolium* Cardot = ***Plagiothecium neckeroideum*** var. ***myurum***
*Plagiothecium laxum* Sakurai = ***Plagiothecium cavifolium***
*Plagiothecium longicaule* Sakurai = ***Plagiothecium neckeroideum***
*Plagiothecium longisetum* var. *brevinerve* Iisiba = ***Plagiothecium longisetum***
*Plagiothecium lucens* Sauter ex Rabenhorst = ***Plagiothecium cavifolium***
*Plagiothecium luridum* (Molendo) Molendo & Lorentz = ***Plagiothecium laetum***
*Plagiothecium maedae* Sakurai = ***Plagiothecium neckeroideum***
*Plagiothecium magufuki* Sakurai = ***Plagiothecium japonicum***
*Plagiothecium matsumarae* S. Okamura = ***Plagiothecium euryphyllum***
*Plagiothecium mauiense* Broth. = ***Plagiothecium longisetum***
*Plagiothecium neckeroideum* var. *angustatum* Cardot = ***Plagiothecium neckeroideum***
*Plagiothecium neckeroideum* var. *argenteus* Dixon = ***Plagiothecium euryphyllum***
*Plagiothecium neckeroideum* var. *sikkimense* Renauld & Cardot = ***Plagiothecium*** ***neckeroideum*** var. ***myurum***
*Plagiothecium neglectum* Mönkm. = ***Plagiothecium nemorale***
*Plagiothecium neglectum* subsp. *platyphyllum* (Mönk.) Szafran = ***Plagiothecium*** ***platyphyllum***
*Plagiothecium neglectum* subsp. *platyphyllum* fo. *fontana* (Mönk.) Jedl. = ***Plagiothecium*** ***platyphyllum***
*Plagiothecium niitakayamae* Toyama = ***Plagiothecium neckeroideum*** var. ***niitakayamae***
*Plagiothecium nipponense* Sakurai = ***Plagiothecium neckeroideum***
*Plagiothecium nitellum* Wilson ex Braithw. = ***Plagiothecium curvifolium***
*Plagiothecium obtusifolium* (Turner) J. J. Amann = ***Plagiothecium denticulatum*** var. ***obtusifolium***
*Plagiothecium orthocladium* Schimp. = ***Plagiothecium cavifolium*** var. ***orthocladium***
*Plagiothecium orthothecioides* (Meyl.) Jedl. = ***Plagiothecium ruthei***
*Plagiothecium otii Sakurai = **Plagiothecium cavifolium***
*Plagiothecium pallidum* S. Okamura = ***Plagiothecium euryphyllum***
*Plagiothecium podperae* Jedl. (Jedl.) = ***Plagiothecium cavifolium***
*Plagiothecium pseudolaetum* var. *japonicum* Cardot = ***Plagiothecium curvifolium***
*Plagiothecium pseudosylvaticum* Warnst. = ***Plagiothecium ruthei***
*Plagiothecium ptychocarpum* Thér. & Dixon = ***Plagiothecium cavifolium***
*Plagiothecium rigens* Broth. = ***Plagiothecium cavifolium***
*Plagiothecium roeseanum* (Hampe) = ***Plagiothecium cavifolium***
*Plagiothecium roeseanum* fo. *angustirete* (Warnst.) Jedl. = ***Plagiothecium cavifolium***
*Plagiothecium roeseanum* fo. *flagellaceum* (Warnst.) Mönk. = ***Plagiothecium cavifolium***
*Plagiothecium roeseanum* fo. *gracile* (Breidl.) Jedl. = ***Plagiothecium cavifolium***
*Plagiothecium roeseanum* fo. *heterophyllum* (Warnst.) Jedl. = ***Plagiothecium cavifolium***
*Plagiothecium roeseanum* fo. *propaguliferum* R. Rute = ***Plagiothecium cavifolium***
*Plagiothecium roeseanum* var. *gracile* Breidl. = ***Plagiothecium cavifolium***
*Plagiothecium roeseanum* var. *gracilescens* E. Bauer = ***Plagiothecium cavifolium***
*Plagiothecium roeseanum* var. *julaceum* Cardot = ***Plagiothecium cavifolium***
*Plagiothecium roeseanum* var. *flagellaceum* Warnst. = ***Plagiothecium cavifolium***
*Plagiothecium roeseanum* var. *orthocladium* (Schimp.) Limpr. = ***Plagiothecium cavifo-*** ***lium*** var. ***orthocladium***
*Plagiothecium roeseanum* var. *propaguliferum* R. Ruthe = ***Plagiothecium cavifolium***
*Plagiothecium roesei* Milde = ***Plagiothecium cavifolium***
*Plagiothecium rosei* Schimp. = ***Plagiothecium cavifolium***
*Plagiothecium rufovirescens* Stirt. = ***Plagiothecium denticulatum***
*Plagiothecium ruthei* var. *gracile* Meyl. = ***Plagiothecium ruthei***
*Plagiothecium ruthei* var. *pseudosylvaticum* (Warnst.) Warnst. = ***Plagiothecium ruthei***
*Plagiothecium ruthei* var. *rupestris* R. Ruthe = ***Plagiothecium ruthei***
*Plagiothecium ruthei* var. *subundulatum* R. Ruthe ex Warnst. = ***Plagiothecium ruthei***
*Plagiothecium sakuraii* Reimers = ***Plagiothecium cavifolium***
*Plagiothecium sandbergii* Renauld & Cardot = ***Plagiothecium denticulatum*** var. ***obtusifolium***
*Plagiothecium saxicola Sakurai = **Plagiothecium nemorale***
*Plagiothecium silvaticum* Bruch & Schimp. in Lindb. = ***Plagiothecium*** ***sylvaticum***
*Plagiothecium silvaticum* var. *latifolium* Cardot = ***Plagiothecium nemorale***
*Plagiothecium silvaticum* var. *nemorale* (Mitt.) Par. = ***Plagiothecium nemorale***
*Plagiothecium silvaticum* var. *rhynchostegioides* Cardot = ***Plagiothecium nemorale***
*Plagiothecium solutans* Mol. ex Warnst. = ***Plagiothecium curvifolium***
*Plagiothecium splendens* Schimp. ex Cardot = ***Plagiothecium euryphyllum***
*Plagiothecium splendens* var. *brevirameum* Cardot = ***Plagiothecium euryphyllum***
*Plagiothecium splendens* var. *minus* Cardot = ***Plagiothecium euryphyllum***
*Plagiothecium splendens* var. *paraphylliferum* Sakurai = ***Plagiothecium euryphyllum***
*Plagiothecium splendens* var. *punctatum* Sakurai = ***Plagiothecium euryphyllum***
*Plagiothecium stoloniferum* Velen. = ***Plagiothecium ruthei***
*Plagiothecium subdenticulatum* Correns = ***Plagiothecium ruthei***
*Plagiothecium sublaetum* (Lindb.) Lindb. = ***Plagiothecium laetum***
*Plagiothecium succulentum* fo. *lignicolum* Jedl. = ***Plagiothecium succulentum*** fo. ***propaguliferum***
*Plagiothecium succulentum* var. *fontanum* (Schiffn.) Riehm. = ***Plagiothecium*** ***platyphyllum***
*Plagiothecium succulentum* var. *longifolium* Mönk = ***Plagiothecium succulentum*** fo. ***propaguliferum***
*Plagiothecium sullivantiae* (Schimp. ex Sull.) A.Jaeger = ***Plagio*** ***thecium cavifolium***
*Plagiothecium sylvaticum* (Brid.) Schimp. = ***Plagiothecium denticulatum***
*Plagiothecium sylvaticum auct. non.* (Brid.) Bruch & Schimp. = ***Plagiothecium nemorale***
*Plagiothecium sylvaticum auct. non. Hypnum sylvaticum* Brid. = ***Plagiothecium nemo-*** ***rale***
*Plagiothecium sylvaticum* subsp. *roesei* Lindb. (Kindb.) = ***Plagiothecium cavifolium***
*Plagiothecium sylvaticum* subsp. *succulentum* (Wils.) J.J. Amann & Meyl. = ***Plagiothecium succulentum***
*Plagiothecium sylvaticum* var. *auritum* Kern = ***Plagiothecium ruthei***
*Plagiothecium sylvaticum* var. *cavifolium* Jur. = ***Plagiothecium cavifolium***
*Plagiothecium sylvaticum* var. *cryptarum* (Renauld & Hérib.) P.Syd. = ***Plagiothecium*** ***cavifolium***
*Plagiothecium sylvaticum* var. *flavescens* Warnst. = ***Plagiothecium platyphyllum***
*Plagiothecium sylvaticum* var. *fluitans* Podp. = ***Plagiothecium platyphyllum***
*Plagiothecium sylvaticum* var. *fontanum* Schiffn. = ***Plagiothecium platyphyllum***
*Plagiothecium sylvaticum* var. *latifolium* Cardot = ***Plagiothecium nemorale***
*Plagiothecium sylvaticum* var. *laxum* Molendo = ***Plagiothecium cavifolium***
*Plagiothecium sylvaticum* var. *monoricum* Breidl. in Limpr. = ***Plagiothecium ruthei*** var. ***rupincola***
*Plagiothecium sylvaticum* var. *myurum* Molendo = ***Plagiothecium cavifolium***
*Plagiothecium sylvaticum* var. *neglectum* (Mönk.) F. Koppe = ***Plagiothecium nemorale***
*Plagiothecium sylvaticum* var. *nemorale* (Mitt.) Paris ≡ ***Plagiothecium nemorale***
*Plagiothecium sylvaticum* var. *nervosum* Renauld = ***Plagiothecium platyphyllum***
*Plagiothecium sylvaticum* var. *orthocladium* (Schimp.) Schimp. = ***Plagiothecium cavi-*** ***folium*** var. ***orthocladium***
*Plagiothecium sylvaticum* var. *phyllorhizans* Spruce = ***Plagiothecium platyphyllum***
*Plagiothecium sylvaticum* var. *platyphyllum* (Mönk) F. Koppe = ***Plagiothecium***
***platyphyllum***
*Plagiothecium sylvaticum* var. *pseudoneckeroideum* Schiffn. = ***Plagiothecium ruthei***
*Plagiothecium sylvaticum* var. *pseudo-roeseanum* Cardot = ***Plagiothecium cavifolium***
*Plagiothecium sylvaticum* var. *rhynchostegioides* Cardot = ***Plagiothecium nemorale***
*Plagiothecium sylvaticum* var. *rivulare* Debat = ***Plagiothecium nemorale***
*Plagiothecium sylvaticum* var. *robustum* Roell. = ***Plagiothecium ruthei***
*Plagiothecium sylvaticum* var. *roeseanum* (Schimp.) A. W. H. Walther & Molendo = ***Plagiothecium cavifolium***
*Plagiothecium sylvaticum* var. *squarrosum* Kind. = ***Plagiothecium platyphyllum***
*Plagiothecium sylvaticum* var. *succulentum* (Wilson) Spruce = ***Plagiothecium succu-*** ***lentum***
*Plagiothecium sylvaticum* var. *sullivantiae* (Sull.) Ren. & Card. = ***Plagiothecium cavi-*** ***folium***
*Plagiothecium takahashii* Sakurai = ***Plagiothecium cavifolium***
*Plagiothecium trichodeum* Stirt. = ***Plagiothecium denticulatum***
*Plagiothecum ruthei* var. *subjulaceum* Warnst. = ***Plagiothecium cavifolium***
*Plagiothecium ruthei* var. *orthothecioides* Meyl. = ***Plagiothecium platyphyllum***
*Rectithecium piliferum* (Sw.) Hadenäs & Huttunen = ***Plagiothecium piliferum***
*Saviczia obtusissima* (Broth.) Abramova & I. I. Abramov = ***Plagiothecium obtusissi-*** ***mum***
*Plagiothecium watanabei* Dixon = ***Plagiothecium euryphyllum***
*Stereodon denticulatus* (Hedw.) Mitt. = ***Plagiothecium denticulatum***
*Stereodon nemoralis* Mitt. ≡ ***Plagiothecium nemorale***
*Stereodon sylvaticus* (Brid.) Brid. = ***Plagiothecium denticulatum***
*Struckia argentata* (Mitt.) Müll. Hal. = ***Plagiothecium argentatum***
*Struckia argentata* var. *enervis* (Broth.) B. C. Tan, W. R. Buck & Ignatov = ***Plagiothe-*** ***cium enerve***
*Struckia enervis* (Broth.) Ignatov, T. J. Kop. & D. G. Long = ***Plagiothecium enerve***

## Appendix B

Checklist of the *Plagiothecium* taxa in Eurasia. Under each taxon, a list of countries and islands on which it was recorded is given along with relevant literature. The taxa are arranged alphabetically under phylogenetically based sectional classification of *Plagiothecium* established by Wynns [7] and Wynns et al. [23], with appropriate changes used in this manuscript.

***Plagiothecium*** Schimp. sect. ***Plagiothecium*** Type: *Hypnum denticulatum* Hedw. Species Muscorum Frondosorum 237. 1801.

1. ***Plagiothecium conostegium*** Herzog 1916. Bibiotheca Botanica 87: 154. f. 73: a–d.
  **JAP** [119].
2. ***Plagiothecium denticulatum*** (Hedw.) Schimp. in BSG 1851. Bryologia Europea 5: 190, 501 (Table VIII).
  **ALB** [9,48]; **AND** [9,47,48,51,52]; **ARM** [32,53]; **AUT** [7,9,48,54]; **AZE** [32]; **AZO** [47]; **BGM** [7,9,48,62]; **BAN** [59,60]; **BUL** [9,48,49,68]; **BIH** [9,47,48,49]; **BLR** [9,32,48,61,228]; **CHI** [9,48]; **SWI** [9,48,176,177]; **CHN** [77,78,79,80]; **COR** [9,47,48,62]; **CZE** [7,9,48,84]; **GER** [7,9,48,95,96,98]; **DEN** [7,9,30,48,87]; **SPA** [9,47,48,51,167]; **EST** [9,32,48,88]; **FIN** [7,9,28,48,91,92]; **FRA** [9,47,48,94]; **FRO** [7,9,48,89,90]; **GRB** [7,9,25,48,109]; **GEO** [32]; **GRC** [9,47,48,49]; **CRO** [9,47,48,49]; **HUN** [9,48,99]; **IND** [103]; **IRE** [9,25,48,109]; **IRN** [45,110]; **IRQ** [106,107]; **ICE** [9,48,102]; **ITA** [9,48,114,115]; **JAP** [33,34,78,118]; **KAZ**; **KGZ** [32]; **KOS** [9]; **SKO** [78,86]; **LAV** [9,32,48,124]; **LIE** [9,48,126]; **LTU** [9,32,48,127]; **LUX** [9,48,129,130]; **MDR** [9,47,131]; **MKD** [9,47,48,49,145]; **MNE** [9,47,48,49]; **MON** [139]; **NET** [9,48,143,144]; **NOR** [7,9,48]; **NEP** [142]; **PAK** [149,150,151]; **POL** [7,9,48,154]; **NKO** [78,86]; **POR** [9,47,48,51,155]; **ROM** [9,48,49,158]; **RUS** [32,48,159]; **SAR** [9,48]; **SIC** [9,47,48]; **SVA** [9,48,173]; **SRB** [9,47,48,49]; **SVK** [9,48,164]; **SVN** [9,47,48,49]; **SWE** [7,9,48,174,175]; **TZK** [32]; **TUR** [9,45,47,48,49,180,181]; **UKR** [9,32,48,183,185].
3. ***Plagiothecium denticulatum*** var. ***affine*** Warnst. 1906. Kryptogamenflora der Mark Brandenburg, Laubmoose 822, 838: f. 1.
  **GER** [7].
4. ***Plagiothecium denticulatum*** var. ***obtusifolium*** (Turner) Moore 1873. Proceedings of the Royal Irish Academy 1: 424.
  **BUL [9,47,48]**; **SWI** [7]; **CHN** [7,79,80]; **CZE** [7,9,48,84]; **GER** [7]; **SPA** [47,48,167]; **FIN** [7,9,48]; **FRA** [7,9,47,48,94]; **GRB** [9,25,48,109]; **HUN** [7]; **IRE** [9,25,48,109]; **IRN** [43,45,110]; **ICE** [7]; **ITA** [9,47,48,115,116,117]; **JAP** [33,118]; **KOS** [9,120]; **LUX** [9,48,129,130]; **NEP** [7]; **POL** [9,48,154]; **RUS** [7,32,48,162]; **SVN** [9,48]; **SWE** [7,9,48,174,175]; **TUR** [9,45,47,48,181]; **UKR** [9,32,48].
5. ***Plagiothecium platyphyllum*** Mönk. 1927. Die Laubmoose Europas 866. f. 207b.
  **AND** [9,52]; **AUT** [7,9,48,54]; **BUL** [9,47,48,49,68]; **SWI** [9,48,176,177]; **CHN** [77,79,80]; **COR** [9,47,48,62]; **CZE** [7,9,48,84]; **GER** [7,9,48,95,97,98]; **DEN** [30]; **SPA** [9,47,48,51,167]; **FIN** [7,9,28,48,91,92,93]; **FRA** [9,47,48,78,94]; **GRB** [7,9,25,48]; **GEO** [32]; **GRC** [9,48]; **CRO** [9,83]; **HUN** [7,9,48,99]; **IRE** [9,25,48,109]; **IRN** [43,45,110]; **ITA** [9,47,48,114,117]; **JAP** [78]; **KOS** [9,120]; **SKO** [78]; **LTU** [9,128]; **LUX** [9,48,129,130]; **MKD** [9,47,48,49]; **MNE** [9,47,48,49]; **NOR** [7,9,48]; **POL** [9,48,154]; **NKO** [78]; **POR** [9,156]; **ROM** [9,48,49,158]; **RUS** [32,48,159]; **SRB** [9,47,48,49]; **SVK** [9,48,164]; **SVN** [9,47,48,49]; **SWE** [7,9,48,174,175]; **TUR** [9,43,45,47,48,49,180,181]; **UKR** [9,32,48,184].
6. ***Plagiothecium ruthei*** Limpr. 1877. Die Laubmoose Deutschlands, Oesterreichs und der Schweiz 3: 271.
  **AUT** [9,48,54]; **BUL** [47,68]; **BLR** [9,48]; **SWI** [7,9,48,176,177]; **CHN** [77]; **CZE** [9,48,84]; **GER** [7,9,48,95,96,98]; **DEN** [7,9,30,48,87]; **SPA** [9,47,48,167]; **EST** [9,32,48,88]; **FIN** [7,9,48,91,92]; **FRA** [9,47,48,94]; **GRB** [9,25,48,109]; **HUN** [9,48,99]; **IRE** [25,109]; **ITA** [47,48,114]; **JAP** [33,118]; **LAV** [9,32,48,124]; **LIE** [9,48,126]; **LTU** [9,32,48,127]; **LUX** [9,48,117,129,130]; **NET** [7,9,48,143,144]; **POL** [7,9,48,154]; **ROM** [9,48]; **RUS** [32,48,159]; **SVK** [9,48,164]; **SWE** [9,48,174,175]; **UKR** [9,32,48,185].
7. ***Plagiothecium ruthei*** var. ***rupincola*** Limpr. 1897 Die Laubmoose Deutschland Oesterreich und der Schweiz 3: 273.
  **AUT; CZE; GER; FRA; NOR; SWE** [7].

***Plagiothecium*** sect. ***Orthophyllum*** Jedl. Spisy Prír. Fak. Masarykovy Univ. 308: 35. 1948.

8. ***Plagiothecium angusticellum*** G. J. Wolski & P. Nowicka-Krawczyk 2020. PLOS ONE 15(3): e0230237.
  **CZE; EST; HUN; LAV; LTU; POL** [27].
9. ***Plagiothecium cavifolium*** (Brid.) Z. Iwats. 1970. Jounral of the Hattori Botanical Labolatory 33: 360.
  **ALB** [9,48]; **AND** [9,52]; **AUT** [9,48,54]; **AZE** [32]; **BGM** [9,48,62]; **BUL** [9,47,48,49,68]; **BIH** [9,47,48,49]; **BHU** [63,64]; **BLR** [9,32,48,61,228]; **SWI** [7,9,48,176,177]; **CHN** [7,64,77,78,79,80]; **COR** [9,47,48,62]; **CZE** [7,9,48,84]; **GER** [7,9,48,95,96,98]; **DEN** [7,9,30,48,87]; **SPA** [9,47,48,51,167]; **EST** [9,32,48,88]; **FIN** [9,28,48,91,92]; **FRA** [7,9,47,48,94]; **FRO** [9,48,89,90]; **GRB** [9,25,48,109]; **GEO** [32]; **GRC** [9,47,48,49]; **CRO** [9,47,48,49]; **HUN** [7,9,48,99]; **IND** [64,103]; **IRE** [9,25,48,109]; **IRN** [111]; **ICE** [7,9,48,102]; **ITA** [9,47,48,114,115,116,117]; **JAP** [7,33,34,64,78,118]; **KAZ** [32]; **SKO** [64,78]; **LAO** [78]; **LAV** [7,9,32,48,124]; **LIE** [9,48]; **LTU** [9,32,48,127]; **LUX** [9,48,129,130]; **MOL** [9,32,48]; **MKD** [9,47,48,49,145]; **MNE** [9,47,48,49]; **MON** [139]; **NET** [9,48,143,144]; **NOR** [9,48]; **NEP** [64,142]; **PAK** [148,149,151]; **POL** [7,9,48,154]; **NKO** [64,78]; **POR** [9,47,48,51,155]; **ROM** [9,48,49,158]; **RUS** [7,48,64,159]; **SIC** [9,47,48]; **SRB** [9,47,48,49]; **SVK** [9,48,164]; **SVN** [9,47,48,49]; **SWE** [9,48,174,175]; **TUR** [9,45,47,48,49,181]; **TAI** [178]; **UKR** [9,32,48,183,185].
10. ***Plagiothecium cavifolium*** var. ***orthocladium*** (Schimp.) Z. Iwats. 1970 Jounral of the Hattori Botanical Labolatory 33: 371.
  **FIN**; **FRO**; **SWE** [7].
11. ***Plagiothecium cochleatum*** Dixon 1938. Notes on the moss collections of the Royal Botanic Garden, Edinburgh. Notes from the Royal Botanic Garden, Edinburgh 19: 299. f. 12.
  **IND** [7,104].
12. ***Plagiothecium japonicum*** Salurai 1949. Botanical Magazine (Tokyo) 62: 112. f. 1.
  **JAP** [7].
13. ***Plagiothecium longisetum*** Lindb. 1872. Contributio ad Floram Cryptogamam Asiae Boreali-Orientalis 232.
  **AUT; AZO; BGM; SWI; CHN; GER; DEN; SPA; EST; FIN; FRA; GRB; GEO; IND; IRN; JAP; MDR; NEP; NOR; POL; RUS; SWE; TUR** [27].
14. ***Plagiothecium nemorale*** (Mitt.) A. Jaeger 1878. Bericht über die Thätigkeit der St. Gallischen Naturwissenschaftlichen Gesellschaft 1876-77: 451 (Gen. Sp, Musc. 2: 1269).
  **ALB** [9,47,48,49]; **AND** [9,47,48,51,52]; **ARM** [32,53]; **AUT** [9,27,48,54]; **AZE** (Ignatov et al. 2006) [32]; **AZO** [9,47,48,55,56,57]; **BGM** [9,27,48,62]; **BUL** [9,47,48,49,68]; **BIH** [9,47,48,49]; **BHU** [7,63]; **BLR** [9,32,48,61,228]; **CNY** [9,47,48]; **CHI** [9,48]; **SWI** [9,48,176,177]; **CHN** [7,27,66,77,78,79,80]; **COR** [9,47,48,62]; **CZE** [7,9,27,48,84]; **GER** [7,9,27,48,95,96,98]; **DEN** [7,9,27,30,48,87]; **SPA** [9,47,48,51,167]; **EST** [9,27,32,48,88]; **FIN** [9,28,48,91,92,93]; **FRA** [7,9,27,47,48,94]; **FRO** [90]; **GRB** [7,9,25,27,48,109]; **GEO** [27,32]; **GRC** [9,47,48,49]; **CRO** [47,48,82]; **HUN** [7,9,27,48,99]; **IND** [7,27,103]; **IRE** [9,25,48,109]; **IRN** [7,27,43,45,111]; **ITA** [7,9,27,47,48,114,115,116,117]; **JAP** [7,27,33,34,66,78,118]; **KOS** [9,120]; **SKO** [7,66,78,86]; **LAV** [9,27,32,48,124]; **LIE** [9,48,126]; **LTU** [9,27,32,48,127]; **LUX** [9,48,129,130]; **MDR** [9,47,48,51,131]; **MKD** [9,47,48,49,145]; **MYA** [72]; **MNE** [9,47,48,49]; **NET** [9,48,143,144]; **NOR** [9,27,48]; **NEP** [7,141,142]; **PAK** [150,151]; **PHI** [152,153]; **POL** [9,27,48,154]; **NKO** [7,66,78,85,86]; **POR** [9,27,47,48,51,155]; **ROM** [9,48,49,158]; **RUS** [7,32,48,66,78,159]; **SAR** [9,47,48]; **SIC** [9,47,48]; **SRB** [9,47,48,49]; **SVK** [9,27,48,164]; **SVN** [9,47,48,49,165]; **SWE** [7,9,27,48,174,175]; **TUR** [7,9,27,43,45,47,48,49,180,181]; **TAI** [66,78,178]; **UKR** [9,32,48,183,185]; **VIE** [189].
15. ***Plagiothecium rhizophyllum*** Sakurai 1932. Botanical Magazine (Tokyo) 46: 501.
  **CHN** [7].
16. ***Plagiothecium succulentum*** (Wilson) Lindb. 1865. Botaniska Notiser 1865: 143.
  **ALB** [9,48]; **AND** [52]; **AUT** [9,48,54]; **AZO** [9,47,48,55,56,57]; **BGM** [9,48,62]; **BUL**; **BIH** [9,47,48,49]; **BLR** [9,48]; **CNY** [9,47,48,74]; **CHI** [9,48]; **SWI** [9,48,176,177]; **CHN** [77,79,80]; **COR** [9,47,48,62]; **CZE** [7,9,48,84]; **GER** [7,9,48,95,96,98]; **DEN** [7,9,30,48,87]; **SPA** [9,47,48,51,167]; **EST** [9,32,48,88]; **FIN** [9,28,48,91]; **FRA** [9,47,48,78,94]; **FRO** [7,9,48,89,90]; **GRB** [9,25,48,109]; **GEO** (Ignatov et al. 2006) [32]; **GRC** [9,47,48,49]; **HUN** [9,48,99]; **IRE** [9,25,48,109]; **IRN** [45,110]; **ICE** [9,48,102]; **ITA** [9,47,48,114,116,117]; **SKO** [78]; **KOS** [9,120]; **LAV** [9,32,48,124]; **LTU** [9,32,48,127]; **LUX** [9,48,129,130]; **MDR** [9,47,48,51,131,132]; **MKD** [9,146]; **MNE**; **NOR** [9,48]; **NET** [9]; **POL** [9,48,154]; **NKO** [78]; **POR** [9,47,48,51,155]; **ROM** [9,48,49,158]; **RUS** [32,48,162]; **SRB** [9,47,48,49]; **SVK** [9,48,164]; **SVN** [9,47,48,49]; **SWE** [7,9,48,174,175]; **TUR** [9,45,47,48,49,181]; **UKR** [9,32,48,184,185].
17. ***Plagiothecium succulentum*** fo. ***propaguliferum*** E. Bauer 1902. Deutsche Botanische Monatsschrift 20: 2.
  **AUT; CZE; GER; DEN; LAV** [7].

***Plagiothecium*** sect. ***Leptophyllum*** Jedl. Spisy Prír. Fak. Masarykovy Univ. 308: 23. 1948.

18. ***Plagiothecium berggrenianum*** Frisvoll 1981. Lindbergia 7: 96. f. 2: a–i.
  **RUS** [7,26,32,48,159]; **SVA** [7,9,48,171,172,173].
19. ***Plagiothecium curvifolium*** Schlieph. ex Limpr. 1897. Die Laubmoose Deutschlands, Oesterreichs und der Schweiz 3: 269.
  **ALB**; **AUT** [9,48]; **BGM** [9,48,62]; **BUL** [9,47,48]; **BIH** [9,47,48,49]; **CHI** [9,48]; **SWI** [9,48,177]; **CHN** [77,79,80]; **COR** [9,47,48,62]; **CZE** [7,9,48,84]; **GER** [7,9,26,48,95,98]; **DEN** [7,9,30,48,87]; **SPA** [9,47,48,51,167]; **EST** [9,32,48,88]; **FIN** [9,28,48,91,92]; **FRA** [9,47,48,94]; **GRB** [9,25,48,109]; **GRC** [9,47,48,49]; **CRO** [9,83]; **HUN** [9,48,100]; **IRE** [9,25,48,109]; **ITA** [9,47,48,116,117]; **JAP** [34]; **KOS** [9,120]; **LAV** [7,9,32,48,124]; **LIE** [9,48,126]; **LTU** [9,32,48,127]; **LUX** [9,48,129,130]; **MKD** [9,47,48]; **MNE** [9,47,48,49]; **NET** [9,143]; **NOR** [9,48]; **POL** [7,9,26,48,154]; **ROM** [9,48,49,158]; **RUS** [26,32,48,161,162]; **SRB** [9,47,48,49]; **SVK** [7,9,48,164]; **SVN** [9,47,48,49]; **SWE** [9,48,174,175]; **TUR** [9,45,47,48,49,181]; **UKR** [9,26,32,48,185].
20. ***Plagiothecium curvifolium*** fo. ***julaceum*** Clum. & E. Bauer 1915. Musci Europaei Exsiccati 27: 1307.
  **SWI [7]**.
21. ***Plagiothecium laetum*** Schimp. in BSG 1851. Bryologia Europaea 5: 185, 495 (Table II).
  **ALB** [9,50]; **AND** [9,47,48,51,52]; **AUT** [9,26,48]; **AZE** [32]; **BGM** [7,9,48,62]; **BUL** [9,47,48,49,68]; **BIH** [9,26,47,48,49]; **BLR** [9,32,48,61,228]; **SWI** [9,48,176,177]; **CHN** [77,79,80]; **CZE** [7,9,26,48,84]; **GER** [7,9,26,48,95,96,98]; **DEN** [7,9,30,48,87]; **SPA** [9,47,48,51,167]; **EST** [9,32,48,88]; **FIN** [7,9,26,28,48,91,92]; **FRA** [9,47,48,94]; **GRB** [9,25,48,109]; **GEO** [32]; **GRC** [9,47,48,49]; **HUN** [9,26,48,99]; **IRE** [9,25,48,109]; **IRN** [43,45,110]; **ITA** [9,47,48,114,115,116,117]; **JAP** [33,78,118]; **KAZ**; **KGZ** [32]; **KOS** [9,120]; **SKO** [78]; **LAV** [9,32,48,124]; **LIE** [9,48,126]; **LTU** [9,32,48,127]; **LUX** [9,48,129,130]; **MDR** [47,51,132]; **MNE** [9,47,48,49]; **MON** [139]; **NET** [9,48,143,144]; **NOR** [9,26,48]; **POL** [7,9,26,48,154]; **NKO** [78]; [47,48,51]; **ROM** [9,48,49,158]; **RUS** [7,26,32,48,159]; **SRB** [9,47,48,49]; **SVK** [9,48,164]; **SVN** [9,47,48,49,165]; **SWE** [7,9,26,48,174,175]; **TUR** [7,45,47,181]; **TAI** [66]; **UKR** [9,32,48,183,185].
22. ***Plagiothecium laetum*** var. ***tenellum*** (Schimp.) Warnst. 1906. Kryptogamenflora der Mark Brandenburg, Laubmoose 835.
  **BGM [7]**.
23. ***Plagiothecium latebricola*** Wilson ex Schimp. in BSG 1851. Bryologia Europaea 5: 184, 494 (Table I).
  **AUT** [9,48,54]; **BGM** [7,9,48,62]; **BLR** [9,32,48,61,228]; **SWI** [48]; **CHN** [76,77,79,80]; **CZE** [9,48,84]; **GER** [9,48,95,96,98,222]; **DEN** [7,9,30,48,87]; **EST** [9,32,48,88]; **FIN** [7,9,28,48,91,92,93]; **FRA** [9,47,48]; **GRB** [7,9,25,48,109]; **GEO** [32]; **HUN** [9,101]; **IRE** [9,25,48,109]; **ITA** [9,47,48,114,117]; **JAP** [33,34,118]; **KGZ** [32]; **LAV** [9,32,48,124]; **LTU** [9,32,48,127]; **SRL** [170]; **LUX** [9,48,129,130]; **NET** [7,9,143,144]; **NOR** [9,48]; **PAK** [150,151]; **POL** [9,48,154]; **POR** [9,47,48,155]; **ROM** [9,48,49,158]; **RUS** [32,48,159]; **SRB** [9,47,48,49]; **SVK** [9,48,164]; **SWE** [9,48,174,175]; **TUR** [43,45,47,180,181]; **UKR** [9,32,48,185].
24. ***Plagiothecium rossicum*** Ignatov & Ignatova 2019. Arctoa 28: 28-45.
  **POL**; **RUS** [9,26].
25. ***Plagiothecium svalbardense*** Frisvoll 1996. Norsk Polarinstitutt Skrifter 198: 103.
  **RUS** [26,32,160,162]; **SVA** [7,9,26,48,173]; **SWE** [7,26].

***Plagiothecium*** sect. ***Pseudo-Neckera*** (Kindb.) J.T. Wynns 2015

26. ***Plagiothecium decoratum*** J. T. Wynns 2015. Molecular phylogeny and systematic revision of the pleurocarpous moss genus *Plagiothecium*. PhD Thesis, University of Copenhagen: 126.
  **BHU; NEP** [7].
27. ***Plagiothecium neckeroideum*** Schimp. in BSG 1851. Bryologia Europea 5: 194, 505 (Table XII).
  **AUT [7,9,48,54]**; **BOR** [65,66]; **BHU** [63]; **SWI** [7,9,48,176,177]; **CHN** [7,66,77,78,79,80]; **CZE** [9,48,84]; **GER** [7,9,48,95,97,98]; **IDN** [105]; **IND** [78,103]; **JAP** [7,33,34,66,78,118]; **SKO** [78]; **MLY** [133]; **NEP** [7,142]; **PHI** [7,66,152,153]; **NKO** [78]; **ROM** [9,48,49,158]; **RUS** [32,66,78,159]; **SIN** [133]; **SUM** [66,105]; **SVN** [9,47,48,49,166]; **THA** [66,72,179]; **TAI** [66,178]; **UKR** [9,32,48,184].
28. ***Plagiothecium neckeroideum*** fo. ***exile*** J. T. Wynns 2015. Molecular phylogeny and systematic revision of the pleurocarpous moss genus *Plagiothecium*. PhD Thesis, University of Copenhagen: 210.
  **NEP** [7].
29. ***Plagiothecium neckeroideum*** var. ***javense*** M. Fleisch. 1920. Die Musci der Flora von Buitenzorg 4: 1168. f. 194.
  **IDN** [7]; **PHI** [152,153]; **SUM** [105].
30. ***Plagiothecium neckeroideum*** var. ***myurum*** Molendo 1875. Jahres-Bericht de Naturhistirischen Vereins in Passau 10: 234.
  **BHU; CHN, IND; NEP** [7].
31. ***Plagiothecium neckeroideum*** var. ***niitakayamae*** (Toyama) Z. Iwats. 1970. Journal of the Hattori Botanical Laboratory 33: 354.
  **CHN** [7,79,80]; **JAP** [29,118]; **PHI** [153]; **TAI** [66,77,178].
32. ***Plagiothecium neckeroideum*** fo. ***parvum*** J. T. Wynns 2015. Molecular phylogeny and systematic revision of the pleurocarpous moss genus *Plagiothecium*. PhD Thesis, University of Copenhagen: 213.
  **TAI** [7].
33. ***Plagiothecium noricum*** Molendo ex Limpr. 1897. Die Laubmoose Deutschlands, Oesterreichs und der Schweiz 3: 257.
  **AUT** [7]; **MYA** [140].
34. ***Plagiothecium subglaucum*** Thwaites & Mitt. 1873. Journal of the Linnean Society, Botany 13: 321.
  **SRL** [7,168,169]; **MYA** [140].

***Plagiothecium*** sect. ***Lycambium*** Jedl. 1948. Spisy Prír. Fak. Masarykovy Univ. 308: 10. 1948.

35. ***Plagiothecium fallax*** Cardot & Thér. 1902. Proceedings of the Washington Academy of Sciences 4: 336. pl. 22: f. 4.
  **JAP**; **RUS** [7].
36. ***Plagiothecium undulatum*** (Hedw.) Schimp. in BSG 1851. Bryologia Europea 5: 195, 506 (Table XIII).
  **AUT** [9,48,54]; **AZE** [32]; **BGM** [9,48,62]; **BUL** [9,47,48,49,68]; **BIH** [47,48,49]; **BLR** [9,32,48,228]; **CHI** [9,48]; **SWI** [9,48,176,177]; **CHN** [77,79]; **CZE** [9,48,84]; **GER** [7,9,48,95,96,98]; **DEN** [7,9,30,48,87]; **SPA** [9,47,48,51,167]; **EST** [9,32,48,88]; **FIN** [9,28,48,91,92]; **FRA** [7,9,47,48,94]; **FRO** [7,9,48,89,90]; **GRB** [7,9,25,48,109]; **CRO** [9,47,48,49]; **HUN** [9,48,99,100]; **IRE** [9,25,48,109]; **IRN** [43,45,110]; **ITA** [9,47,48,114,115,116,117]; **LAV** [9,32,48,124]; **LIE** [9,48,126]; **LTU** [9,32,48,127]; **LUX** [9,48,129,130]; **NET** [9,48,143,144]; **NOR** [9,48]; **POL** [7,9,48,154]; **POR** [9,47,48,51]; **ROM** [9,48,49,158]; **RUS** [32,48,159]; **SIC** [9,47,48]; **SRB** [9,47,48,49]; **SVK** [9,48,164]; **SVN** [9,47,48,49]; **SWE** [7,9,48,174,175]; **TUR** [43,45,47,180,181]; **UKR** [9,32,48,184].

***Plagiothecium*** sect. ***Saviczia*** (Abramova & I. I. Abramov) Z. Iwats. J. Hattori Bot. Lab. 33: 341. 1970.

37. ***Plagiothecium euryphyllum*** (Cardot & Thér.) Z. Iwats. 1970. Jounral of the Hattori Botanical Labolatory 33: 348.
  **BHU [64]**; **CHN** [7,64,77,79,80]; **IND** [64]; **JAP** [7,33,34,64,78,118]; **MYA** [140]; **SKO**; **NKO** [64,78]; **RUS** [32,161,162]; **TAI** [78,178]; **VIE** [72,188,189].
38. ***Plagiothecium obtusissimum*** Broth. 1921. Oefversigt at Förhandlingar, Finska Vetenskaps-Societeten 62A(9): 45.
  **JAP** [7,33,34]; **RUS** [32,159].

***Plagiothecium*** sect. ***Struckia*** (Müll. Hal.) J.T. Wynns 2015

39. ***Plagiothecium argentatum*** (Mitt.) Q. Zuo 2011. Journal of Bryology 33(3): 227.
  **CHN [7,36]**; **IND**; **NEP** [7].
40. ***Plagiothecium enerve*** (Broth.) Q. Zuo 2011. Journal of Bryology 33(3): 227.
  **CHN** [7,36]; **RUS** [7].

***Plagiothecium*** sect. ***Rectithecium*** (Hedenäs & Huttunen) J. T. Wynns 2015

41. ***Plagiothecium piliferum*** (Sw.) Schimp. in BSG 1851. Bryologia Europea 5: 186, 496 (Table III).
  **AND** [9,52]; **SWI** [9,48,176,177]; **CHN** [77,78,79,80]; **COR** [9,47,48,62]; **DEN** [30]; **SPA** [9,47,48,51,167]; **FIN** [7,9,28,48,91,92]; **FRA** [9,47,48,78,94]; **GRB** [7,9,25,48,109]; **IRE** [25,109]; **ITA** [9,47,48,114,115,117]; **JAP** [33,78]; **SKO** [78]; **LAV** [9,125]; **NOR** [7,9,48]; **NKO** [78]; **POR** [9,47,48,51,155]; **ROM** [9,48,49,158]; **RUS** [7,32,48,78,159]; **SAR** [9,47,48]; **SVN** [9,47,48,49,165]; **SWE** [7,9,48,174,175]; **TUR** [47,182]; **UKR** [9,32,48,184,185].
