# Supplementary material for: The Genus Plagiothecium Schimp. (Plagiotheciaceae, Bryophyta) in Eurasia: An Annotated Checklist with Distribution and Ecological Data
Source: Plants (Basel). 2021 Apr 26;10(5):868. doi: 10.3390/plants10050868 (PMC8145931; doi:10.3390/plants10050868)
Supplement: Supplementary file 1 [file plants-10-00868-s001.zip › plants-1156055-supplementary.pdf]

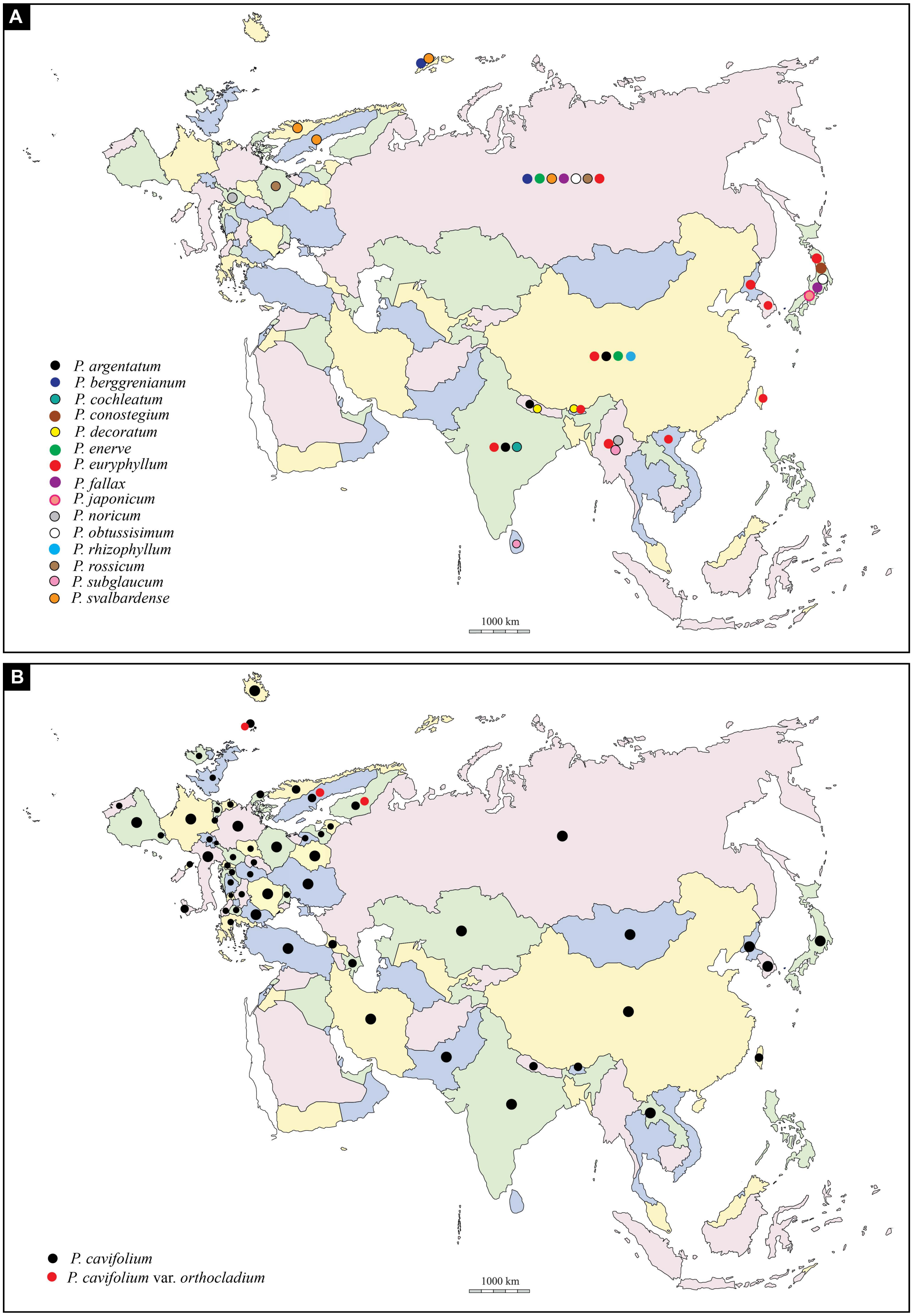

Figure S1. Maps showing the distribution of *Plagiothecium* taxa in Eurasia per country/region. **A.** The distribution of *P. argentatum*, *P. berggrenianum*, *P. cochleatum*, *P. conostegium*, *P. decoratum*, *P. enerve*, *P. euryphyllum*, *P. fallax*, *P. japonicum*, *P. noricum*, *P. obtussisimum*, *P. rhizophyllum*, *P. rossicum*, *P. subglaucum*, and *P. svalbardense*. **B.** The distribution of *P. cavifolium* and *P. cavifolium* var. *orthocladium*.

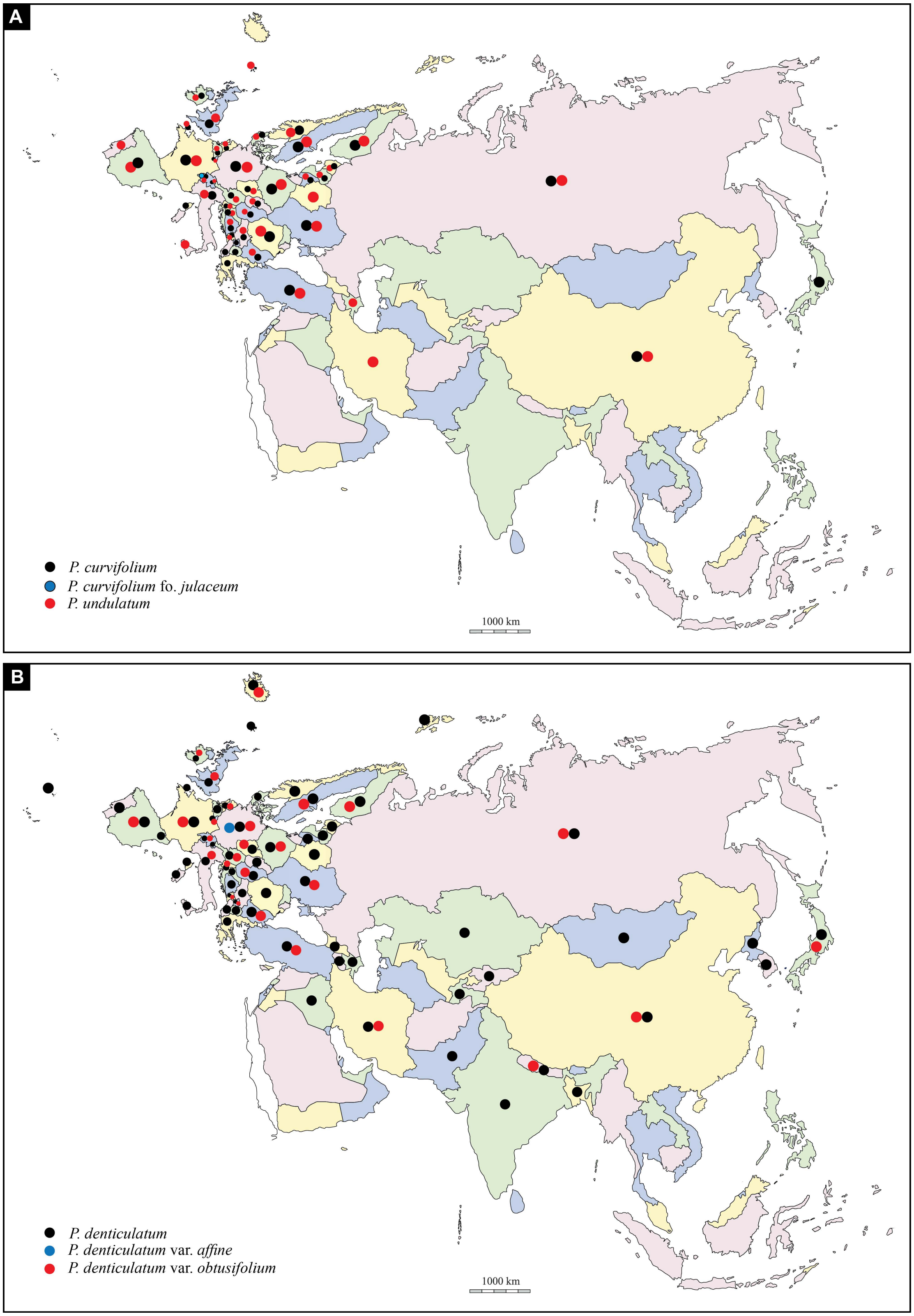

Figure S2. Maps showing the distribution of *Plagiothecium* taxa per country/region. **A.** The distribution of *P. curvifolium*, *P. curvifolium* fo. *julaceum*, and *P. undulatum*. **B.** The distribution of *P. denticulatum*, *P. denticulatum* var. *affine*, and *P. denticulatum* var. *obtusifolium*.

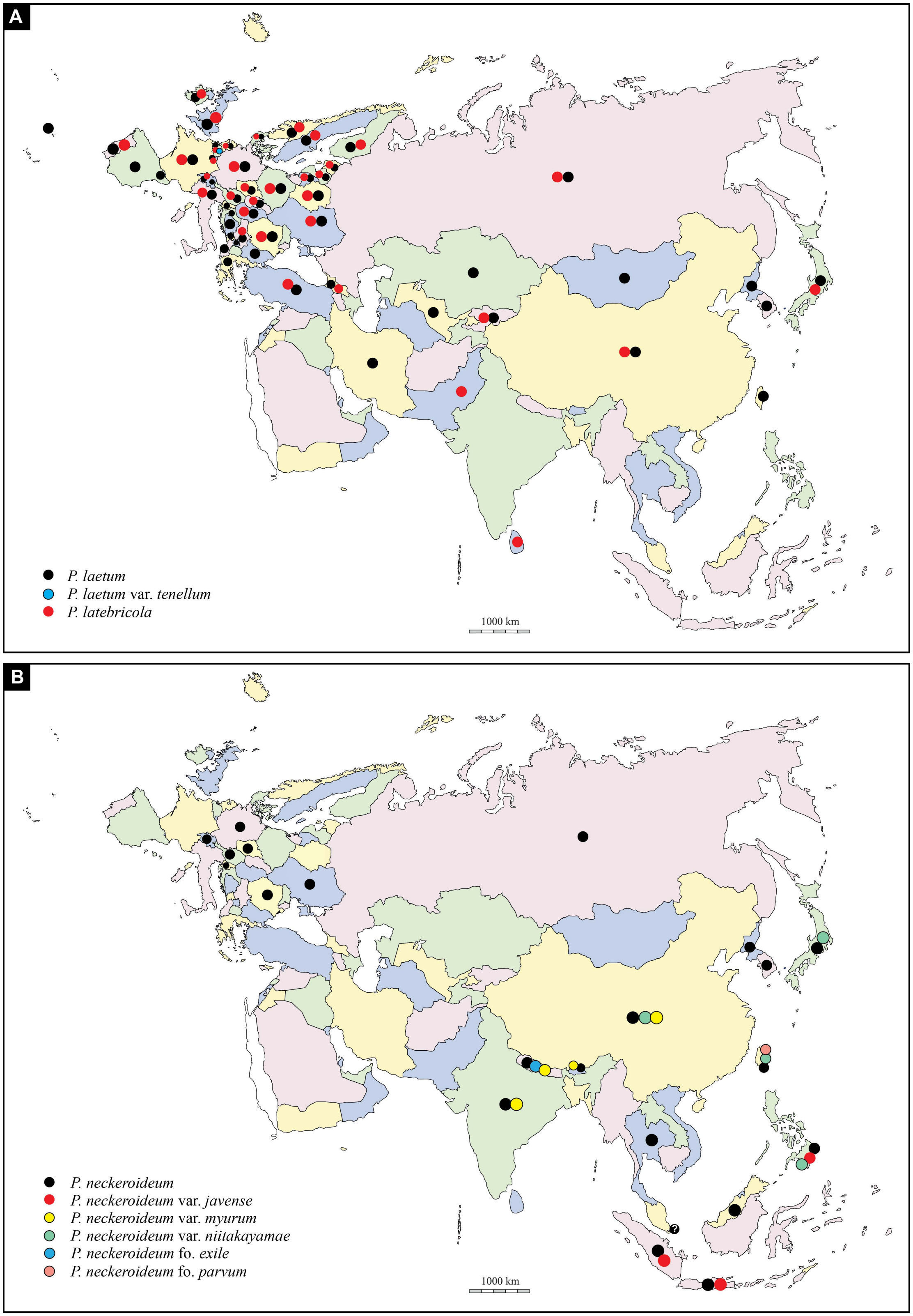

Figure S3. Maps showing the distribution of *Plagiothecium* taxa per country/region. **A.** The distribution of *P. laetum*, *P. laetum* var. *tenellum*, and *P. latebricola*. **B**. The distribution of *P. neckeroideum*, *P. neckeroideum* var. *javense*, *P. neckeroideum* var. *myurum*, *P. neckeroideum* var. *niitakayamae*, *P. neckeroideum* fo. *exile*, and *P. neckeroideum* fo. *parvum*.

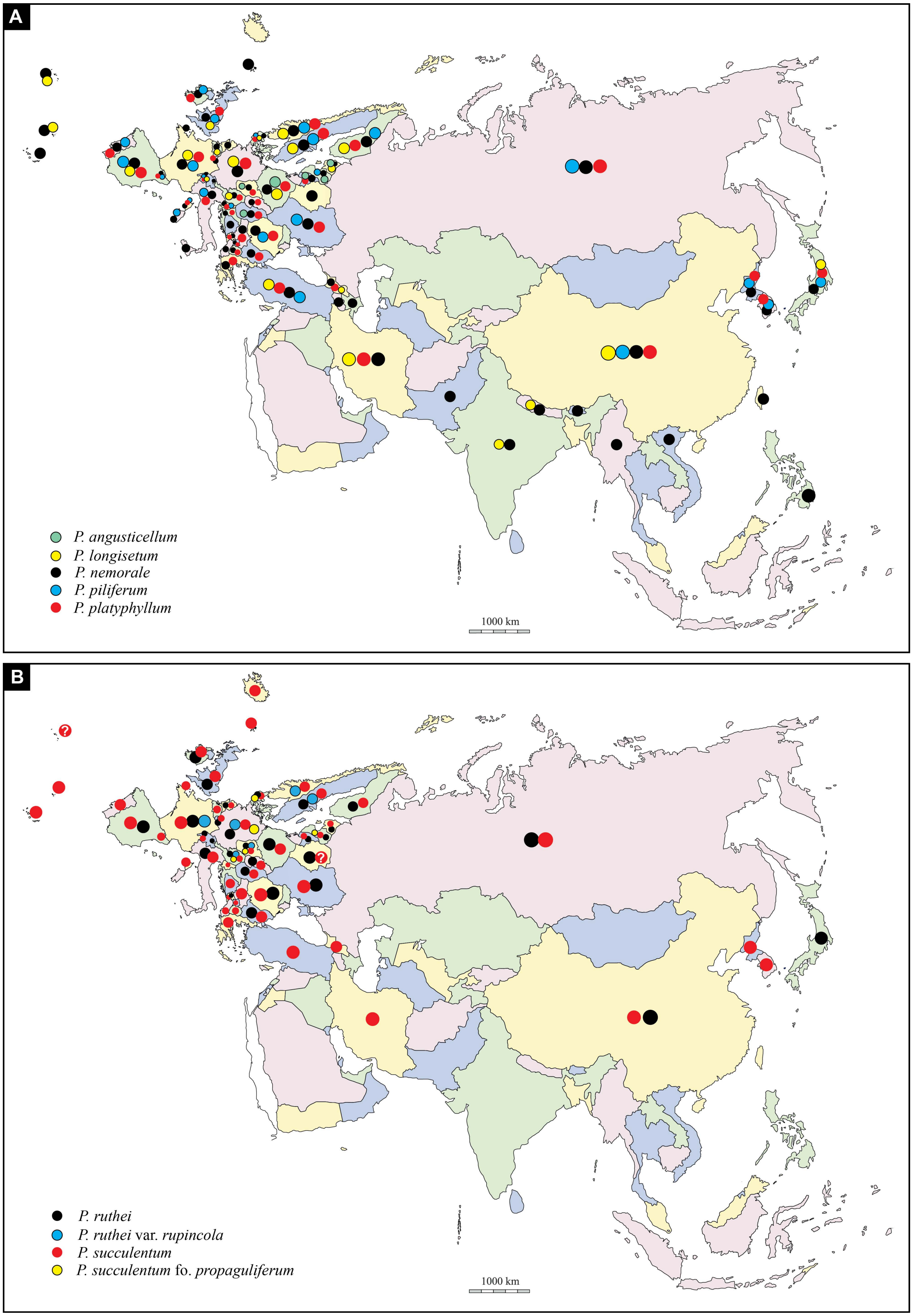

Figure S4. Maps showing the distribution of *Plagiothecium* taxa per country/region. **A.** The distribution of *P. angusticellum*, *P. longisetum*, *P. nemorale*, *P. piliferum*, and *P. platyphyllum*. **B.** The distribution of *P. ruthei*, *P. ruthei* var. *rupicola*, *P. succulentum*, and *P. succulentum* fo. *propaguliferum*.
